# Eco-Friendly Synthesis, Characterization and Biological Evaluation of Some Novel Pyrazolines Containing Thiazole Moiety as Potential Anticancer and Antimicrobial Agents

**DOI:** 10.3390/molecules23112970

**Published:** 2018-11-14

**Authors:** Mastoura M. Edrees, Sraa Abu- Melha, Amirah M. Saad, Nabila A. Kheder, Sobhi M. Gomha, Zeinab A. Muhammad

**Affiliations:** 1 Department of Chemistry, Faculty of Science, King Khalid University, Abha 61413, Saudi Arabia; sabomlba@kku.edu.sa; 2 Department of Organic Chemistry, National Organization for Drug Control and Research (NODAR), Giza 12311, Egypt; zeinab.a.muhammad@gmail.com; 3 Department of Pharmaceutical Chemistry, Faculty of Pharmacy, King Khalid University, Abha 61441, Saudi Arabia; ammaalqahtani@kku.edu.sa (A.M.S.); nabila.abdelshafy@gmail.com (N.A.K.); 4 Department of chemistry, Faculty of Science, Cairo University, Giza 12613, Egypt

**Keywords:** pyrazolines, thiazoles, hydrazonoyl halides, antimicrobial activity, anticancer activity

## Abstract

The one-pot synthesis of a series of pyrazoline derivatives containing the bioactive thiazole ring has been performed through a 1,3-dipolar cycloaddition reaction of *N*-thiocarbamoylpyrazoline and different hydrazonoyl halides or α-haloketones in the presence of DABCO (1,4-diazabicyclo[2.2.2] octane) as an eco-friendly catalyst using the solvent-drop grinding method. The structure of the synthesized compounds was elucidated using elemental and spectroscopic analyses (IR, NMR, and Mass). The activity of these compounds against human hepatocellular carcinoma cell line (HepG2) was tested and the results showed that the pyrazoline **11f**, which has a fluorine substituent, is the most active. The antimicrobial activities of the newly synthesized compounds were determined against two fungi and four bacterial strains, and the results indicated that some of the newly synthesized pyrazolines are more potent than the standard drugs against test organisms.

## 1. Introduction

The majority of antitumor drugs have many serious side effects. Another fundamental problem in chemotherapy is the emergence of cancer cell drug resistance [1]. Also, there is a rapid evolution of drug-resistant pathogens that raise the need for the discovery of new drugs to counterbalance the effects of this resistance. Pyrazolines have been found to possess diverse biological activities such as anticancer [2,3,4], antitumor [5], antioxidant [6], antimicrobial [7,8], antitubercular [9], antimalarial [10], anti-amoebic [11], DPPH radical scavenging, anti-diabetic [12], antiviral [13] and amine oxidase [14]. Also, thiazoles are known to have anticonvulsant [15], antimicrobial [16], anti-inflammatory [17], anticancer [18,19,20], antidiabetic [21], anti-HIV [22], anti-Alzheimer [23], antihypertensive [24], antifungal [25], and antioxidant [26] activities. In view of the above mentioned findings and as a part of our research interest towards developing new ways to synthesize a variety of heterocyclic systems with promising biological and pharmacological activities [27,28,29,30], we present in this research an efficient synthesis of a series of pyrazolines attached to thiazole moiety using *N*-thiocarbamoylpyrazoline [31] and the appropriate hydrazonoyl halides [32,33,34] or α-haloketones in the presence of DABCO under the solvent-drop grinding method [35].

## 2. Results and Discussion

### 2.1. Chemistry

**Scheme 1:**
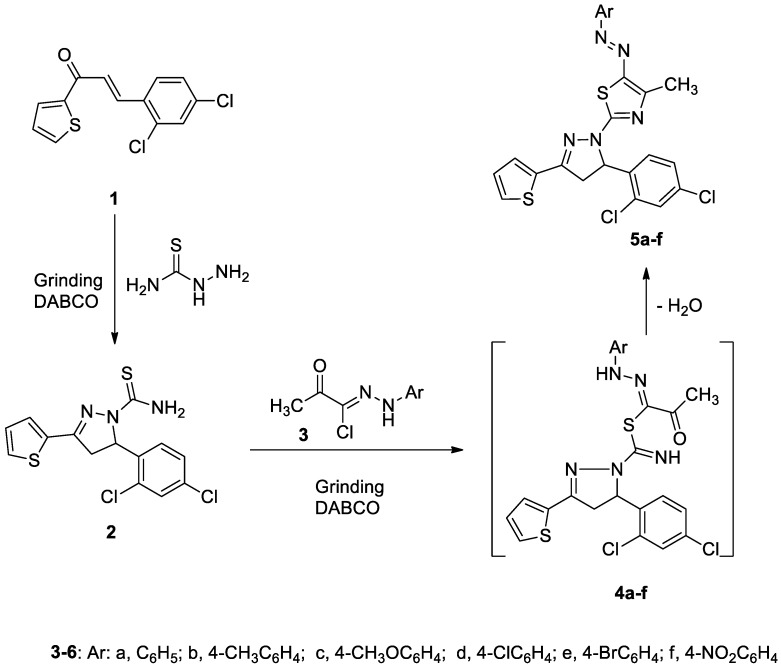
Synthesis of pyrazoline derivatives **5a**–**f**.

*N*-Thiocarbamoylpyrazoline **2** was prepared by cyclization of 3-(2,4-dichlorophenyl)-1-(thiophen-2-yl)prop-2-en-1-one (**1)** [31] with thiosemicarbazide in the presence of DABCO as a basic catalyst using a grinding method at room temperature as shown in Scheme 1. The reaction between the pyrazoline **2** and the appropriate hydrazonoyl halides **3a**–**f** [32,33,34] in the presence of DABCO using the solvent-drop grinding method afforded a series of pyrazolines attached to the bioactive thiazole moiety (Scheme 1). The structures of the synthesized compounds **5a**–**f** were elucidated using elemental and spectroscopic analysis (IR, NMR, and Mass). The ^1^H-NMR of pyrazolines **5a**–**f** showed in each case a singlet signal at *δ* 2.16–2.49 ppm due to the methyl protons, in addition to the expected protons of pyrazoline and aromatic rings. Also, their mass spectra showed in each case a molecular ion peak which agrees with the proposed structures (see the Experimental section). The reaction was assumed to start intially through nucleophilic displacement of the halide to afford intermediate **4**, which underwent intramolecular cyclization and dehydration to afford the final product **5** (Scheme 1).

**Scheme 2:**
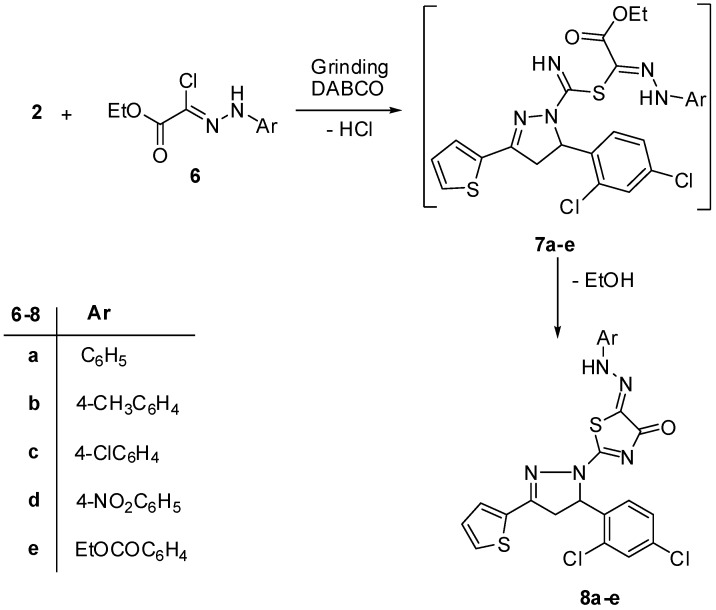
Synthesis of pyrazolylthiazolons **8a**–**e**.

Next, the pyrazoline derivative **2** was reacted with the appropriate hydrazonoyl halides **6a**–**e** [36] under the same experimental conditions to give the pyrazolylthiazolone derivatives **8a**–**e** (Scheme 2). The IR spectra of pyrazolines **8a**–**e** revealed in each case absorption bands at ν = 1649–1686, and 3430–3434 cm^−1^ corresponding to carbonyl and NH-hydrazo groups, respectively. Also, their ^1^H-NMR spectra revealed a singlet signal in the region *δ* 11.17–11.74 ppm due to the NH proton. In addition, their mass spectra showed the expected peaks due to their molecular ions.

The reaction was suggested to proceed through nucleophilic displacement of chloride to give intermediate **7** which underwent elimination of one ethanol molecule to afford the target products **8a**–**e**. 

**Scheme 3:**
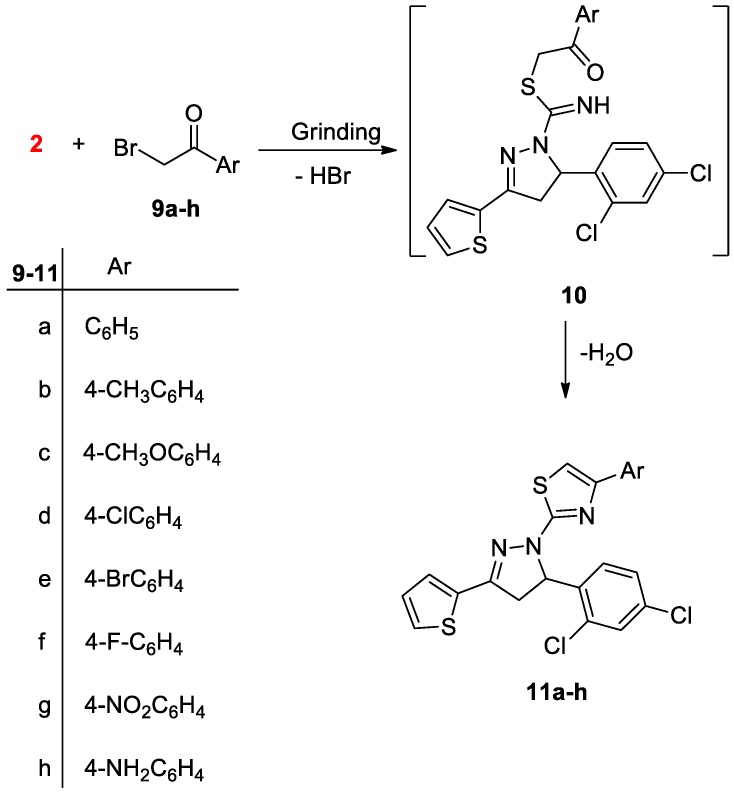
Synthesis of pyrazolylthiazoles **11a**–**h**.

Finally, the reaction between the pyrazoline derivative **2** and appropriate α-haloketones **9a**–**h** afforded pyrazolylthiazole derivatives **11a**–**h** (Scheme 3). The proposed structures are consistent with the analytical and spectroscopic analyses (see the Experimental section).

### 2.2. Pharmacology

#### 2.2.1. Antitumor Activity

The antitumor activity of the products **5a**–**e** and **8a**–**d**, and **11a**–**f** was investigated against human hepatocellular carcinoma cell line (HepG2), in comparison with Cisplatin as the anticancer standard drug [37,38]. IC_50_ (the concentration of test compounds required to kill 50% of cell population) was determined from the dose response curve (Table 1).

**Figure 1:**
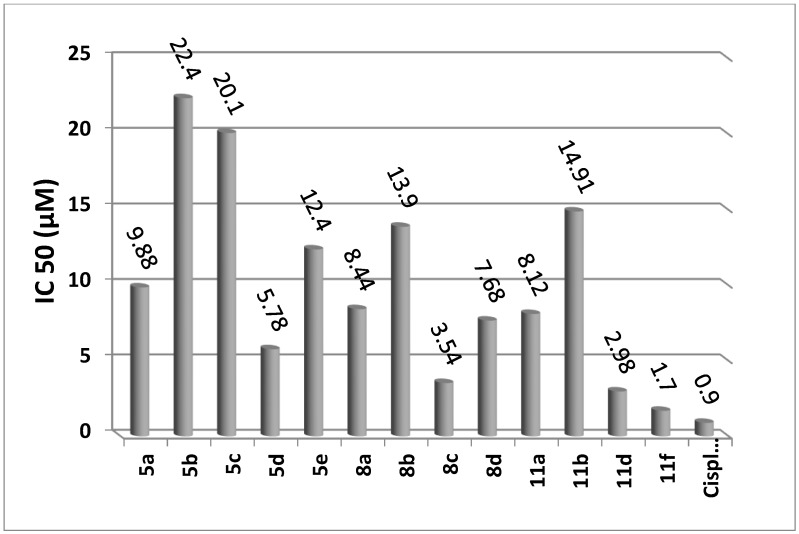
Cytotoxic activities of tested compounds against HepG2.

Figure 1 shows a comparison between the values of IC_50_ of the evaluated pyrazolines against Cisplatin, which is used as a standard drug.

The IC_50_ of cytotoxic activity data (Table 1 and Figure 1) showed that the pyrazoline derivative **11f**, which bears a fluorine substituent had the highest cytotoxic activity compared to Cisplatin. 

From the obtained results, we suggest the following structural requirements for activity:

- The phenyl ring substitution (X) is important for activity.
- The electronic nature of phenyl ring substitution (X) is important.
- Compounds with an electron withdrawing *para*-substituents demonstrate good activity.
- A phenyl ring substituted at the C-4 position by halogens has increased activity.
- A fluorine atom at the *para* position of the phenyl ring optimizes activity.
- Phenyl rings with para alkyl groups show decreased activity.

#### 2.2.2. Evaluation of the Antimicrobial Activity 

The in vitro antimicrobial activities of the newly synthesized compounds and reference drugs were tested by inhibition zone technique [39,40] and minimum inhibitory concentration (MIC), using two fungi: Aspergillus fumigatus (RCMB 002008 (4) and Candida albicans (RCMB 05036), two gram-positive bacteria: *Staphylococcus aureus* (RCMB 010010), and *Bacillus subtilis* (RCMB 010067), two gram-negative bacteria: *Escherichia coli* (RCMB 010052), and *Proteus vulgaris* RCMB 004 (1) ATCC 13315, and the results are depicted in Table 2 and Table 3. The data showed that some of the newly synthesized pyrazolines are able to inhibit the growth of the examined microbes in vitro and some of them are more potent than the standard drugs. In general, the chemical structure of the whole molecule, comprising the nature of the heterocyclic system as well as the type of the substituted function present in the heterocyclic ring structure, has a pronounced effect. All data were recorded as the mean of three replicates with standard deviation (±SD) using the software Excel (Microsoft, New York, NY, USA)

From the screening results, it can be seen that:

- Pyrazoline derivatives **11** have higher activity than **5** and **8** against the tested bacteria and fungi
- Pyrazoline derivatives **11a**, **11b**, **11d**, and **11e** are the most potent compounds against *Aspergillus fumigatus* and they had higher potency than the standard drug, *Ketoconazole*.
- The pyrazoline **11a** exhibited high antifungal activity against *CA*, and is more potent than the standard drug *Ketoconazole*.
- The pyrazoline derivative **11a** is more potent than the reference drug *Gentamycin* against *SA* and *PV*.
- The higher antimicrobial activity of pyrazolyl-thiazole derivative **11a** is due to the phenyl group at position 4 of the thiazole ring.
- Most of the tested compounds have higher activity against gram positive bacteria than gram negative bacteria.
- Compound **5e** gave no action with all the tested species
- Most of the tested compounds have higher activity against bacteria than fungi
- Pyrazoline derivatives **8** have higher activity against *PV* than *EC*

In addition, the minimum inhibitory concentration (MIC) of compounds **5a**–**f**, **8a**–**e** and **11a**–**h** was considered to be the lowest concentration of the tested substance exhibiting no visible growth of the bacteria or fungi on the plate as shown in Table 3. 

The results of minimum inhibitory concentration of the tested compounds **5a**–**f**, **8a**–**e** and **11a**–**h** exhibited that:

- The synthesized pyrazolines showed a broad spectrum of activities with MIC values from 9.77–10,000 µg mL^−1^.
- Compound **11a** is the most active compound against all the tested microorganisms.

## 3. Experimental 

### 3.1. Chemistry 

Melting points were measured on an Electrothermal IA 9000 series digital melting point apparatus (Bibby Sci. Lim. Stone, Staffordshire, UK). IR spectra were recorded in potassium bromide discs on PyeUnicam SP 3300 and Shimadzu FTIR 8101 PC infrared spectrophotometers (Shimadzu, Tokyo, Japan). ^1^H-NMR spectra were recorded on a Varian Mercury VX-300 NMR spectrometer (Varian, Inc., Karlsruhe, Germany) operating at 300 MHz (^1^H-NMR) and run in deuterated dimethylsulfoxide (DMSO-*d_6_*). Chemical shifts were related to that of the solvent. Mass spectra were recorded on a Shimadzu GCMS-QP1000 EX mass spectrometer (Tokyo, Japan) at 70 eV. Elemental analyses were measured by using a German made Elementarvario LIII CHNS analyzer. The biological evaluations of the products were carried out in the medical mycology laboratory of the regional center for mycology and biotechnology of Al-Azhar University, Cairo, Egypt. The 3-(2,4-dichlorophenyl)-1-(thiophen-2-yl)prop-2-en-1-one (**1**) [31], the hydrazonoyl halides **3a** [32,33], **3b**–**f** [34] and **6a**–**e** [36] were prepared as described in the literature.

Synthesis of 5-(2,4-dichlorophenyl)-3-(thiophen-2-yl)-4,5-dihydro-1*H*-pyrazole-1-carbothioamide (**2**). A mixture of 3-(2,4-dichlorophenyl)-1-(thiophen-2-yl)prop-2-en-1-one (**1**) (2.83 g, 10 mmol) and thiosemicarbazide (0.91 g, 10 mmol) was taken in a mortar at room temperature. A catalytic amount of DABCO was added. The reaction mixture was ground by the pestle, under the hood, for 15 min. The reaction mixture was then poured into water, and the solid product was collected by filtration. The crude product was recrystallized from ethanol as yellow crystals, 62% yield, m.p. 189–191 °C; IR (KBr) v_max_ 3041, 2936 (C-H), 1600 (C=N) cm^−1^; ^1^H-NMR (DMSO-*d*_6_) *δ* 3.19 (m, 1H, CH), 3.56 (m, 1H, CH), 5.82 (m, 1H, CH), 7.21–7.85 (m, 6H, Ar-H), 8.47 (s, 2H, NH_2_), ppm; MS *m*/*z* (%) 356 (M^+^, 17), 280 (49), 193 (27), 104 (30), 76 (82), 41 (100). Anal. Calcd for C_14_H_11_Cl_2_N_3_S_2_ (356.29): C, 47.19; H, 3.11; N, 11.79. Found: C, 47.05; H, 3.03; N, 11.58%.

General method for the synthesis of pyrazolylthiazoles **5a**–**f**, **8a**–**e** and **11a**–**f**. A mixture of the appropriate hydrazonoyl chlorides **3** or **6** or α-haloketones **9** (1 mmol) and the pyrazole-1-carbothioamide **2** (0.233 g, 1 mmol) was taken in a mortar at room temperature. A catalytic amount of DABCO was added. The reaction mixture was ground by the pestle under the hood for 10–20 min (monitored through TLC). The reaction mixture was then poured into water, and the solid product was collected by filtration. The crude product was recrystallized from the appropriate solvent to give the corresponding pyrazolylthiazoles **5a**–**f**, **8a**–**e** and **11a**–**f**, respectively. The products together with their physical data are listed below.

*2-(5-(2,4-Dichlorophenyl)-3-(thiophen-2-yl)-4,5-dihydro-1H-pyrazol-1-yl)-4-methyl-5-(phenyldiazenyl)thiazole* (**5a**). Red color, 72% yield, m.p. 135–136 °C (EtOH); IR (KBr) v_max_ 3054, 2917 (C-H), 1601 (C=N) cm^−1^; ^1^H-NMR (DMSO-*d*_6_) *δ* 2.47 (s, 3H, CH_3_), 3.14 (m, 1H, CH), 3.54 (m, 1H, CH), 5.95 (m, 1H, CH), 7.05–8.05 (m, 11H, Ar-H) ppm; ^13^C-NMR NMR (DMSO-*d*_6_) *δ* 16.0 (CH_3_), 43.3 (CH_2_), 56.8 (CH), 122.12, 123.98, 128.34, 128.51, 128.62, 129.19, 129.76, 130.2, 130.3, 130.20, 130.48, 137.26, 41.24, 142.23, 143.15, 144.13, 152.58, 158.26, 164.42 (Ar-C and C=N) ppm; MS *m*/*z* (%) 498 (M^+^, 8), 482 (40), 347 (37), 319 (62), 280 (78), 233 (53), 175 (30), 126 (39), 76 (58), 54 (45), 41 (100). Anal. Calcd for C_2__3_H_17_Cl_2_N_5_S_2_ (498.44): C, 55.42; H, 3.44; N, 14.05. Found: C, 55.65; H, 3.26; N, 13.91%.

*2-(5-(2,4-Dichlorophenyl)-3-(thiophen-2-yl)-4,5-dihydro-1H-pyrazol-1-yl)-4-methyl-5-(p-tolyldiazenyl)thiazole* (**5b**). Red color, 74% yield, m.p. 137–139 °C (EtOH); IR (KBr) v_max_ 3120, 2917 (C-H), 1609 (C=N) cm^−1^; ^1^H-NMR (DMSO-*d*_6_) *δ* 2.16 (s, 3H, CH_3_), 2.29 (s, 3H, CH_3_), 3.05 (m, 1H, CH), 3.57 (m, 1H, CH), 5.99 (m, 1H, CH), 7.07–8.95 (m, 10H, Ar-H) ppm; ^13^C-NMR (DMSO-*d*_6_) *δ* 16.29, 20.31 (CH_3_), 43.0 (CH_2_), 57.93 (CH), 119.30, 121.24, 125.81, 128.17, 128.74, 129.11, 130.38, 130.90, 131.05, 131.62, 134.29, 137.02, 140.34, 140.86, 142.58, 144.06, 157.16, 164.05 (Ar-C and C=N) ppm; MS *m*/*z* (%) 512 (M^+^, 20), 484 (35), 423 (20), 392 (82), 320 (76), 289 (64), 249 (48), 141 (53), 104 (48), 76 (89), 64 (100), 50 (97). Anal. Calcd for C_24_H_19_Cl_2_N_5_S_2_ (512.47): C, 56.25; H, 3.74; N, 13.67. Found: C, 56.45; H, 3.65; N, 13.45%.

*2-(5-(2,4-Dichlorophenyl)-3-(thiophen-2-yl)-4,5-dihydro-1H-pyrazol-1-yl)-5-((4-methoxyphenyl) diazenyl)-4-methylthiazole* (**5c**). Red color, 71% yield, m.p. 130–132 °C (EtOH); IR (KBr) v_max_ 3068, 2925 (C-H), 1593 (C=N) cm^−1^; ^1^H-NMR (DMSO-*d*_6_) *δ* 2.27 (s, 3H, CH_3_), 3.05 (m, 1H, CH), 3.57 (m, 1H, CH), 3.75 (s, 3H, OCH_3_), 6.03 (m, 1H, CH), 6.94–8.59 (m, 10H, Ar-H) ppm; MS *m*/*z* (%) 528 (M^+^, 9), 472 (10), 365 (30), 320 (23), 267 (53), 239 (28), 161 (62), 133 (100), 117 (48), 104 (68), 78 (40), 57 (42). Anal. Calcd for C_24_H_19_Cl_2_N_5_OS_2_ (528.48): C, 54.54; H, 3.62; N, 13.25. Found: C, 54.76; H, 3.45; N, 13.11%.

*5-((4-Chlorophenyl)diazenyl)-2-(5-(2,4-dichlorophenyl)-3-(thiophen-2-yl)-4,5-dihydro-1H-pyrazol-1-yl)-4-methylthiazole* (**5d**). Red color, 67% yield, m.p. 120–122 °C (EtOH); IR (KBr) v_max_ 3081, 2921 (C-H), 1600 (C=N) cm^−1^; ^1^H-NMR (DMSO-*d*_6_) *δ* 2.42 (s, 3H, CH_3_), 3.04 (m, 1H, CH), 3.57 (m, 1H, CH), 6.06 (m, 1H, CH), 7.07–8.94 (m, 10H, Ar-H) ppm; MS *m*/*z* (%) 532 (M^+^, 10), 518 (34), 493 (51), 409 (99), 383 (84), 347 (33), 281 (59), 198 (60), 76 (30), 43 (100). Anal. Calcd for C_23_H_16_Cl_3_N_5_S_2_ (532.89): C, 51.84; H, 3.03; N, 13.14. Found: C, 52.11; H, 2.91; N, 12.92%.

*5-((4-Bromophenyl)diazenyl)-2-(5-(2,4-dichlorophenyl)-3-(thiophen-2-yl)-4,5-dihydro-1H-pyrazol-1-yl)-4-methylthiazole* (**5e**). Red color, 75% yield, m.p. 143–145 ^o^C (Dioxane); IR (KBr) v_max_ 3147, 2969 (C-H), 1604 (C=N) cm^−1^; ^1^H-NMR (DMSO-*d*_6_) *δ* 2.49 (s, 3H, CH_3_), 3.07 (m, 1H, CH), 3.52 (m, 1H, CH), 6.01 (m, 1H, CH), 7.40–8.42 (m, 10H, Ar-H) ppm; MS *m*/*z* (%) 577 (M^+^, 7), 523 (24), 439 (21), 332 (58), 299 (34), 146 (57), 110 (28), 68 (100), 42 (77). Anal. Calcd for C_23_H_16_BrCl_2_N_5_S_2_ (577.34): C, 47.85; H, 2.79; N, 12.13. Found: C, 48.07; H, 2.65; N, 12.01%.

*2-(5-(2,4-Dichlorophenyl)-3-(thiophen-2-yl)-4,5-dihydro-1H-pyrazol-1-yl)-4-methyl-5-((4-nitrophenyl)diazenyl)thiazole* (**5f**). Orange color, 68% yield, m.p. 140–141 °C (Dioxane); IR (KBr) v_max_ 3084, 2938 (C-H), 1605 (C=N) cm^−1^; ^1^H-NMR (DMSO-*d*_6_) *δ* 2.43 (s, 3H, CH_3_), 3.04 (m, 1H, CH), 3.54 (m, 1H, CH), 6.05 (m, 1H, CH), 7.30–7.99 (m, 10H, Ar-H) ppm; MS *m*/*z* (%) 543 (M^+^, 12), 485 (27), 453 (31), 394 (100), 318 (39), 258 (26), 244 (33), 160 (37), 107 (54), 81 (65), 43 (91). Anal. Calcd for C_23_H_16_Cl_2_N_6_O_2_S_2_ (543.44): C, 50.83; H, 2.97; N, 15.46. Found: C, 51.12, H, 2.75; N, 15.27%.

*2-(5-(2,4-Dichlorophenyl)-3-(thiophen-2-yl)-4,5-dihydro-1H-pyrazol-1-yl)-5-(2-phenylhydrazono) thiazol-4(5H)-one* (**8a**). Yellow color, 70% yield, m.p.145–147 °C (EtOH); IR (KBr) v_max_ 3433 (NH), 3093, 2923 (C-H), 1686 (C=O), 1612 (C=N) cm^−1^; ^1^H-NMR (DMSO-*d*_6_) *δ* 3.03 (m, 1H, CH), 3.56 (m, 1H, CH), 5.96 (m, 1H, CH), 6.94–8.69 (m, 11H, Ar-H), 11.74 (s, 1H, NH) ppm; MS *m*/*z* (%) 500 (M^+^, 14), 422 (23), 396 (56), 316 (100), 276 (10), 161 (39), 104 (36), 82 (64), 67 (60), 43 (93). Anal. Calcd for C_22_H_15_Cl_2_N_5_OS_2_ (500.42): C, 52.80; H, 3.02; N, 14.00. Found: C, 53.05; H, 2.87; N, 13.77%.

*2-(5-(2,4-Dichlorophenyl)-3-(thiophen-2-yl)-4,5-dihydro-1H-pyrazol-1-yl)-5-(2-(p-tolyl)hydrazono)thiazol-4(5H)-one* (**8b**). Yellow color, 72% yield, m.p. 163–165 °C (Dioxane); IR (KBr) v_max_ 3434 (NH), 3066, 2922 (C-H), 1649 (C=O), 1612 (C=N) cm^−1^; ^13^C-NMR (DMSO-*d*_6_) *δ* 20.84 (CH_3_), 43.32 (CH_2_), 60.47 (CH), 112.53, 115.07, 118.93, 126.11, 127.76, 127.95, 129.67, 130.13, 130.62, 131.20, 132.27, 133.32, 140.70, 150.36, 151.39, 163.37, 168.10 (Ar-C and C=N), 170.85 (C=O) ppm; ^1^H-NMR (DMSO-*d*_6_) *δ* 2.27 (s, 3H, CH_3_), 3.02 (m, 1H, CH), 3.58 (m, 1H, CH), 5.98 (m, 1H, CH), 7.11–8.31 (m, 10H, Ar-H), 11.66 (s, H, NH) ppm; MS *m*/*z* (%) 514 (M^+^, 20), 502 (50), 469 (39), 439 (44), 320 (84), 294 (34), 158 (38), 119 (40), 77 (100), 42 (31). Anal. Calcd for C_23_H_17_Cl_2_N_5_OS_2_ (514.44): C, 53.70; H, 3.33; N, 13.61. Found: C, 53.55; H, 3.50; N, 13.29%.

*5-(2-(4-Chlorophenyl)hydrazono)-2-(5-(2,4-dichlorophenyl)-3-(thiophen-2-yl)-4,5-dihydro-1H-pyrazol-1-yl)thiazol-4(5H)-one* (**8c**). Yellow color, 70% yield, m.p. 178–180 °C (DMF); IR (KBr) v_max_ 3433 (NH), 3089, 2926 (C-H), 1649 (C=O), 1617 (C=N) cm^−1^; ^1^H-NMR (DMSO-*d*_6_) *δ* 3.05 (m, 1H, CH), 3.52 (m, 1H, CH), 5.97 (m, 1H, CH), 7.08–8.62 (m, 10H, Ar-H), 11.69 (s, 1H, NH) ppm; MS *m*/*z* (%) 534 (M^+^, 12), 514 (44), 456 (21), 432 (16), 327 (38), 316 (63), 297 (100), 239 (26), 135 (31), 95 (72), 68 (43), 43 (59). Anal. Calcd for C_22_H_14_Cl_3_N_5_OS_2_ (534.86): C, 49.40; H, 2.64; N, 13.09. Found: C, 49.62; H, 2.43; N, 13.25%.

*2-(5-(2,4-Dichlorophenyl)-3-(thiophen-2-yl)-4,5-dihydro-1H-pyrazol-1-yl)-5-(2-(4-nitrophenyl) hydrazono)thiazol-4(5H)-one* (**8d**). Yellow color, 74% yield, m.p. 155–156 °C (EtOH); IR (KBr) v_max_ 3432 (NH), 3079, 2924 (C-H), 1653 (C=O), 1592 (C=N) cm^−1^; ^1^H-NMR (DMSO-*d*_6_) *δ* 3.07 (m, 1H, CH), 3.54 (m, 1H, CH), 6.04 (m, 1H, CH), 7.11–8.69 (m, 10H, Ar-H), 11.62 (s, 1H, NH) ppm; MS *m*/*z* (%) 545 (M^+^, 5), 502 (18), 422 (47), 396 (39), 353 (60), 317 (100), 257 (25), 199 (21), 164 (40), 101 (52), 95 (36), 78 (42). Anal. Calcd for C_22_H_14_Cl_2_N_6_O_3_S_2_ (545.41): C, 48.45; H, 2.59; N, 15.41. Found: C, 48.52; H, 2.75; N, 15.33%. 

*Ethyl-4-(2-(2-(5-(2,4-dichlorophenyl)-3-(thiophen-2-yl)-4,5-dihydro-1H-pyrazol-1-yl)-4-oxothiazol-5(4H)-ylidene)hydrazinyl)benzoate* (**8e**). Yellow color, 67% yield, m.p. 168–170 °C (DMF); IR (KBr) v_max_ 3430 (NH), 3079, 2916 (C-H), 1649 (C=O), 1590 (C=N) cm^−1^; ^1^H-NMR (DMSO-*d*_6_) *δ* 1.17 (t, 3H, CH_3_), 3.00 (m, 1H, CH), 3.54 (m, 1H, CH), 4.25 (q, 2H, CH_2_), 5.95 (m, 1H, CH), 7.04–8.19 (m, 10H, Ar-H), 11.17 (s, 1H, NH) ppm: MS *m*/*z* (%) 572 (M^+^, 32), 509 (31), 458 (49), 439 (100), 402 (50), 387 (56), 359 (58), 347 (62), 94 (43), 68 (52), 42 (78). Anal. Calcd for C_25_H_19_Cl_2_N_5_O_3_S_2_ (572.48): C, 52.45; H, 3.35; N, 12.23. Found: C, 52.66; H, 3.17; N, 12.06%.

*2-(5-(2,4-Dichlorophenyl)-3-(thiophen-2-yl)-4,5-dihydro-1H-pyrazol-1-yl)-4-phenylthiazole* (**11a**). Yellow color, 79% yield, m.p. 193–195 °C (Dioxane); IR (KBr) v_max_ 3054, 2920 (C-H), 1609 (C=N) cm^−1^; ^1^H-NMR (DMSO-*d*_6_) *δ* 3.01 (m, 1H, CH), 3.53 (m, 1H, CH), 5.97 (m, 1H, CH), 6.98–7.86 (m, 11H, Ar-H), 8.39 (s, 1H, thiazole-H) ppm; MS *m*/*z* (%) 456 (M^+^, 17), 427 (56), 366 (52), 285 (35), 200 (39), 172 (100), 131 (93), 101 (38), 77 (85). Anal. Calcd for C_22_H_15_Cl_2_N_3_S_2_ (456.41): C, 57.89; H, 3.31; N, 9.21. Found: C, 57.72; H, 3.25; N, 9.10%.

*2-(5-(2,4-Dichlorophenyl)-3-(thiophen-2-yl)-4,5-dihydro-1H-pyrazol-1-yl)-4-(p-tolyl)thiazole* (**11b**). Yellow brown, 78% yield, m.p. 184–186 °C (Dioxane); IR (KBr) v_max_ 3032, 2915 (C-H), 1608 (C=N) cm^−1^; ^1^H-NMR (DMSO-*d*_6_) *δ* 2.27 (s, 3H, CH_3_), 3.01 (m, 1H, CH), 3.47 (m, 1H, CH), 6.03 (m, 1H, CH), 6.95–8.00 (m, 10H, Ar-H), 8.35 (s, 1H, thiazole-H) ppm; ^13^C-NMR (DMSO-*d*_6_) *δ* 19.01 (CH_3_), 43.74 (CH_2_), 60.22 (CH), 103.81, 125.98, 128.21, 128.42, 129.34, 129.52, 129.65, 132.11, 133.25, 133.98, 134.53, 135.53, 136.48, 137.33, 137.44, 144.11, 166.89, 168.12 (Ar-C and C=N) ppm; MS *m*/*z* (%) 468 (M^+^-2, 10), 418 (24), 399 (59), 353 (74), 285 (59), 204 (45), 184 (49), 92 (70), 44 (100). Anal. Calcd for C_23_H_17_Cl_2_N_3_S_2_ (470.44): C, 58.72; H, 3.64; N, 8.93. Found: C, 58.79; H, 3.52; N, 8.75%.

*2-(5-(2,4-Dichlorophenyl)-3-(thiophen-2-yl)-4,5-dihydro-1H-pyrazol-1-yl)-4-(4-methoxyphenyl)thiazole* (**11c**). Yellow color, 71% yield, m.p.168–170 °C (EtOH); IR (KBr) v_max_ 3088, 2931 (C-H), 1591 (C=N) cm^−1^; ^1^H-NMR (DMSO-*d*_6_) *δ* 3.04 (m, 1H, CH), 3.57 (m, 1H, CH), 3.79 (s, 3H, OCH_3_), 5.99 (m, 1H, CH), 6.89–8.37 (m, 10H, Ar-H), 8.42 (s, 1H, thiazole-H) ppm. MS *m*/*z* (%) 486 (M^+^, 13), 420 (19), 245 (23), 170 (54), 92 (100), 83 (16), 72 (5). Anal. Calcd for C_23_H_17_Cl_2_N_3_OS_2_ (486.44): C, 56.79; H, 3.52; N, 8.64. Found: C, 56.73; H, 3.45; N, 8.33%.

*4-(4-Chlorophenyl)-2-(5-(2,4-dichlorophenyl)-3-(thiophen-2-yl)-4,5-dihydro-1H-pyrazol-1-yl)thiazole* (**11d**). Yellow color, 75% yield, m.p. 189–191 °C (Dioxane); IR (KBr) v_max_ 3086, 2925 (C-H), 1611 (C=N) cm^−1^; ^1^H-NMR (DMSO-*d*_6_) *δ* 3.02 (m, 1H, CH), 3.54 (m, 1H, CH), 5.97 (m, 1H, CH), 6.951–8.00 (m, 10H, Ar-H), 8.36 (s, 1H, thiazole-H) ppm. MS *m*/*z* (%) 490 (M^+^, 8), 455 (38), 424 (92), 404 (54), 320 (100), 186 (79), 132 (46), 75 (51). Anal. Calcd for C_22_H_14_Cl_3_N_3_S_2_ (490.86): C, 53.83; H, 2.87; N, 8.56. Found: C, 53.69; H, 3.04; N, 8.39%.

*4-(4-Bromophenyl)-2-(5-(2,4-dichlorophenyl)-3-(thiophen-2-yl)-4,5-dihydro-1H-pyrazol-1-yl)thiazole* (**11e**). Yellow color, 74% yield, m.p. 182–184 °C (Dioxane); IR (KBr) v_max_ 3068, 2920 (C-H), 1611 (C=N) cm^−1^; ^1^H-NMR (DMSO-*d*_6_) *δ* 3.04 (m, 1H, CH), 3.48 (m, 1H, CH), 5.95 (m, 1H, CH), 6.97–8.30 (m, 10H, Ar-H), 8.32 (s, 1H, thiazole-H5) ppm; MS *m*/*z* (%) 535 (M^+^, 11), 524 (43), 511 (94), 472 (92), 432 (100), 404 (93), 352 (47), 312 (48), 184 (41), 76 (75), 57 (51). Anal. Calcd for C_22_H_14_BrCl_2_N_3_S_2_ (535.31): C, 49.36; H, 2.64; N, 7.85. Found: C, 49.29; H, 2.50; N, 7.65%.

*2-(5-(2,4-Dichlorophenyl)-3-(thiophen-2-yl)-4,5-dihydro-1H-pyrazol-1-yl)-4-(4-fluorophenyl)thiazole* (**11f**). 75% yield, m.p. 175–177 °C (EtOH); IR (KBr) v_max_ 3073, 2925 (C-H), 1591 (C=N) cm^−1^; ^1^H-NMR (DMSO-*d*_6_) *δ* 3.11 (m, 1H, CH), 3.51 (m, 1H, CH), 5.91(m, 1H, CH), 7.18-8.35 (m, 10H, Ar-H), 8.42 (s, 1H, thiazole-H) ppm; MS *m*/*z* (%) 474 (M^+^, 14), 436 (33), 408 (45), 399 (60), 368 (100), 313 (89), 264 (56), 143 (31), 68 (59). Anal. Calcd for C_22_H_14_Cl_2_FN_3_S_2_ (474.40): C, 55.70; H, 2.97; N, 8.86. Found: C, 55.65; H, 2.88; N, 8.64%.

*2-(5-(2,4-Dichlorophenyl)-3-(thiophen-2-yl)-4,5-dihydro-1H-pyrazol-1-yl)-4-(4-nitrophenyl)thiazole* (**11g**). Brown color, 82% yield, m.p. 205–207 °C (DMF); IR (KBr) v_max_ 3145, 2934 (C-H), 1590 (C=N) cm^−1^; ^1^H-NMR (DMSO-*d*_6_) *δ* 3.14 (m, 1H, CH), 3.64 (m, 1H, CH), 5.86 (m, 1H, CH), 7.24–8.38 (m, 10H, Ar-H), 8.42 (s, 1H, thiazole-H) ppm; MS *m*/*z* (%) 501 (M^+^, 18), 487 (46), 461 (74), 425 (100), 396 (98), 315 (49), 287 (43), 223 (13), 166 (25), 130 (23). Anal. Calcd for C_22_H_14_Cl_2_N_4_O_2_S_2_ (501.41): C, 52.70; H, 2.81; N, 11.17. Found: C, 52.64; H, 2.73; N, 11.00%.

*4-(2-(5-(2,4-Dichlorophenyl)-3-(thiophen-2-yl)-4,5-dihydro-1H-pyrazol-1-yl)thiazol-4-yl)aniline* (**11h**). Brown color, 78% yield, m.p. 184–186 °C (Dioxane); IR (KBr) v_max_ 3394-3264 (NH_2_), 3147, 2935 (C-H), 1690 (C=N) cm^−1^; ^1^H-NMR (DMSO-*d*_6_) *δ* 3.01 (m, 1H, CH), 3.55 (m, 1H, CH), 6.00 (m, 1H, CH), 6.08 (br s, 2H, NH_2_), 6.95–8.00 (m, 10H, Ar-H), 8.65 (s, 1H, thiazole-H) ppm; MS *m*/*z* (%) 471 (M^+^, 28), 461 (100), 433 (66), 397 (64), 366 (75), 306 (17), 298 (17), 141 (26), 70 (93). Anal. Calcd for C_22_H_16_Cl_2_N_4_S_2_ (471.43): C, 56.05; H, 3.42; N, 11.88. Found: C, 55.99; H, 3.67; N, 11.67%.

### 3.2. Cytotoxic Activity.

The cytotoxic activity of the synthesized compounds was evaluated against the liver Carcinoma (HepG2) cell line at the Regional Center for Mycology and Biotechnology at Al-Azhar University, Cairo, Egypt according to the reported methods [37,38]. For more details, see the supporting information file. 

### 3.3. Antimicrobial Evaluation.

Agar Diffusion Well Method [39,40] was used to determine the antimicrobial activity of the synthesized compounds. For more details, see the supporting information file. 

## 4. Conclusions

We used an eco-friendly method for the synthesis of a novel series of pyrazolines containing the bioactive thiazole moiety. The structures of the newly synthesized compounds were established based on both elemental and spectroscopic analysis. The cytotoxic activity against liver Carcinoma (HepG2) cell line was measured and it showed that the pyrazoline derivatives **11f**, **11d** and **8c** had IC_50_ values of 1.7, 2.98 and 3.54 µM, respectively. The results of the antifungal evaluation revealed that the pyrazolines **11a**, **11b**, **11d**, and **11e** are the most potent compounds against *Aspergillus fumigatus* and the pyrazoline **11a** is more potent than the standard drug *Ketoconazole* against *Candida albicans*. The results of antibacterial evaluation showed that the pyrazoline **11a** is more potent than the standard drug *Gentamycin* against *Staphylococcus aureus* and *Proteus vulgaris*.

## Figures and Tables

**Table 1:** The in vitro inhibitory activity of the synthesized pyrazolines against HepG2 cell line expressed as IC_50_ values (μM) ± standard deviation from three replicates.

Tested Compounds	IC_50_ (μM)
**5a**	9.88 ± 1.8
**5b**	22.40 ± 1.1
**5c**	20.10 ± 2.7
**5d**	5.78 ± 1.8
**5e**	12.4 ± 2.9
**8a**	8.44 ± 1.9
**8b**	13.90 ± 2.1
**8c**	3.54 ± 1.8
**8d**	7.68 ± 1.9
**11a**	8.12 ± 1.2
**11b**	14.91 ± 1.8
**11d**	2.98 ± 1.8
**11f**	1.70 ± 8.2
**Cisplatin**	0.90 ± 1.1

The data are expressed in the form of mean ± standard error.

**Table 2:** Antimicrobial activities of the new pyrazolines **5a**–**f**, **8a**–**e** and **11a**–**h** expressed as inhibition diameter zones in millimeters (mm) based on well diffusion assay.

Sample	Microorganisms					
	FUNGI		Gram Positive Bacteria		Gram Negative Bacteria	
	*AF*	*CA*	*SA*	*BS*	*EC*	*PV*
**5a**	NA	9 ± 0.4	10 ± 0.7	NA	8 ± **0.2**	10 ± 0.7
**5b**	NA	NA	10 ± 0.80	9 ± 0.4	11 ± 0.6	13 ± 0.6
**5c**	10 ± 0.4	NA	13 ± 0.7	12 ± 0.4	11 ± 0.5	10 ± 0.7
**5d**	NA	NA	9 ± 0.2	10 ± 0.3	9 ± 0.4	12 ± 0.9
**5e**	NA	NA	NA	NA	NA	NA
**5f**	NA	NA	12 ± 0.6	13 ± 0.5	12 ± 0.9	15 ± 1.1
**8a**	NA	NA	11 ± 0.7	13 ± 0.7	11 ± 0.8	15 ± 0.9
**8b**	NA	NA	9 ± 0.3	12 ± 0.8	13 ± 0.9	12 ± 0.6
**8c**	NA	NA	10 ± 0.3	13 ± 0.4	15 ± 0.7	14 ± 0.4
**8d**	NA	NA	11 ± 0.6	13 ± 0.7	12 ± 0.4	15 ± 0.5
**8e**	NA	NA	12 ± 0.7	11 ± 0.4	13 ± 0.6	14 ± 0.8
**11a**	28 ± 0.9	23 ± 1.1	25 ± 1.4	18 ± 0.6	17 ± 0.6	28 ± 1.4
**11b**	21 ± 0.8	14 ± 0.6	12 ± 0.5	11 ± 0.6	12 ± 0.6	14 ± 0.7
**11c**	12 ± 0.8	10 ± 0.4	NA	12 ± 0.7	13 ± 0.9	11 ± 0.6
**11d**	20 ± 1.2	NA	10 ± 0.7	9 ± 0.4	10 ± 0.4	12 ± 0.3
**11e**	25 ± 0.7	NA	9 ± 0.5	NA	NA	10 ± 0.3
**11f**	NA	NA	10 ± 0.4	11 ± 0.6	12 ± 0.9	11 ± 0.3
**11g**	NA	12 ± 0.7	11 ± 0.5	14 ± 0.6	15 ± 0.7	16 ± 0.9
**11h**	NA	NA	12 ± 0.9	13 ± 0.4	14 ± 0.8	13 ± 0.5
***Ketoconazole***	17 ± 0.4	20 ± 0.8	-	-	-	-
***Gentamycin***	-	-	24 ± 1.2	26 ± 0.7	30 ± 0.9	25 ± 0.8

NA. No activity, data are expressed in the form of mean of inhibition zone diameter (mm) for test compounds and performed in triplicate ± SD; AF (*Aspergillus fumigatus* (RCMB 002008 (4)), *CA* (*Candida albicans* (RCMB 05036), *SA* (*Staphylococcus aureus* CMB 010010)), *BS* (*Bacillus subtilis* (RCMB 010067)), *EC* (*Escherichia coli* (RCMB 010052)), *PV* (*Proteus vulgaris* RCMB 004 (1) ATCC 13315).

**Table 3:** Antimicrobial activities of the newly synthesized pyrazolines **5a**–**f**, **8a**–**e** and **11a**–**h** were expressed as MIC in µg/mL.

Sample	Microorganisms					
	FUNGI		Gram Positive Bacteria		Gram Negative Bacteria	
	*AF*	*CA*	*SA*	*BS*	*EC*	*PV*
**5a**	-	10,000	5000	-	10,000	5000
**5b**	-	-	10,000	10,000	5000	625
**5c**	10,000	-	625	1250	2500	5000
**5d**	-	-	5000	2500	5000	312.5
**5e**	-	-	-	-	-	-
**5f**	-	-	625	312.5	625	156.25
**8a**	-	-	625	156.25	1250	39.06
**8b**	-	-	5000	312.5	625	625
**8c**	-	-	2500	625	156.25	312.5
**8d**	-	-	2500	156.25	312.5	156.25
**8e**	-	-	312.5	2500	78.13	156.25
**11a**	312.5	625	9.77	78.13	78.13	19.53
**11b**	156.25	1250	2500	1250	1250	312.5
**11c**	2500	-	-	1250	625	2500
**11d**	625	-	50,000	10,000	10,000	1250
**11e**	312.5	-	5000	-	-	5000
**11f**	-	-	5000	2500	625	2500
**11g**	-	-	5000	156.25	78.13	78.13
**11h**	-	-	2500	625	312.5	625

## Supplementary Materials

The following are available online, Methods of the cytotoxic, and antimicrobial evaluation, Figures (Figures S1–S5) of mean zone of inhibition, and the NMRs of the new synthesized compounds.
