# Supplementary material for: Eco-Friendly Synthesis, Characterization and Biological Evaluation of Some Novel Pyrazolines Containing Thiazole Moiety as Potential Anticancer and Antimicrobial Agents
Source: Molecules. 2018 Nov 14;23(11):2970. doi: 10.3390/molecules23112970 (PMC6278264; doi:10.3390/molecules23112970)
Supplement: Supplementary file 1 [file molecules-23-02970-s001.pdf]

Eco-Friendly Synthesis, Characterization and Biological Evaluation of Some Novel Pyrazolines Containing Thiazole Moiety as Potential Anticancer and Antimicrobial Agents

Mastoura M. Edrees[1,2,*], Sraa Abu- Melha[1], Amirah M. Saad[3], Nabila A. Kheder[4], Sobhi M. Gomha[4], and Zeinab A Muhammad[2]

General method for the evaluation of Cytotoxic Effects of certain Chemical compound

Mammalian cell lines: MCF-7 cells (human breast cancer cell line), HepG-2 cells (human Hepatocellular carcinoma) and HCT-116 (colon carcinoma) were obtained from VACSERA Tissue Culture Unit.

Chemicals Used: Dimethyl sulfoxide (DMSO), crystal violet and trypan blue dye were purchased from Sigma (St. Louis, Mo., USA).

Fetal Bovine serum, DMEM, RPMI-1640, HEPES buffer solution, L-glutamine, gentamycin and 0.25% Trypsin-EDTA were purchased from Lonza.

Crystal violet stain (1%): It composed of 0.5% (w/v) crystal violet and 50% methanol then made up to volume with ddH_2O and filtered through a Whatmann No.1 filter paper.

Cell line Propagation:

The cells were propagated in Dulbecco's modified Eagle's medium (DMEM) supplemented with 10% heat-inactivated fetal bovine serum, 1% L-glutamine, HEPES buffer and 50µg/ml gentamycin. All cells were maintained at 37ºC in a humidified atmosphere with 5% CO_2 and were subcultured two times a week.

Cytotoxicity evaluation using viability assay [36,37]: For cytotoxicity assay, the cells were seeded in 96-well plate at a cell concentration of 1×10^4 cells per well in 100µl of growth medium. Fresh medium containing different concentrations of the test sample was added after 24 h of seeding. Serial two-fold dilutions of the tested chemical compound were added to confluent cell monolayers dispensed into 96-well, flat-bottomed microtiter plates (Falcon, NJ, USA) using a multichannel pipette. The microtiter plates were incubated at 37ºC in a humidified incubator with 5% CO_2 for a

period of 24 h. Three wells were used for each concentration of the test sample. Control cells were incubated without test sample and with or without DMSO. The little percentage of DMSO present in the wells (maximal 0.1%) was found not to affect the experiment. After incubation of the cells at 37°C, for 24 h, the viable cells yield was determined by a colorimetric method.

In brief, after the end of the incubation period, media were aspirated and the crystal violet solution (1%) was added to each well for at least 30 minutes. The stain was removed and the plates were rinsed using tap water until all excess stain is removed. Glacial acetic acid (30%) was then added to all wells and mixed thoroughly, and then the absorbance of the plates were measured after gently shaken on Microplate reader (TECAN, Inc.), using a test wavelength of 490 nm. All results were corrected for background absorbance detected in wells without added stain. Treated samples were compared with the cell control in the absence of the tested compounds. All experiments were carried out in triplicate. The cell cytotoxic effect of each tested compound was calculated. The optical density was measured with the microplate reader (SunRise, TECAN, Inc, USA) to determine the number of viable cells and the percentage of viability was calculated as [(ODt/ODc)]x100% where ODt is the mean optical density of wells treated with the tested sample and ODc is the mean optical density of untreated cells. The relation between surviving cells and drug concentration is plotted to get the survival curve of each tumor cell line after treatment with the specified compound. The 50% inhibitory concentration (IC_{50}), the concentration required to cause toxic effects in 50% of intact cells, was estimated from graphic plots of the dose response curve for each conc. using Graphpad Prism software (San Diego, CA. USA).

Method of antimicrobial evaluation.

All microbial strains were provided from culture collection of the Regional Center for Mycology and Biotechnology (RCMB), Al-Azhar University, Cairo, Egypt. The antimicrobial activity was investigated on a newly synthesized compound in order to increase the selectivity of these derivatives towards test microorganisms using the agar diffusion method using Mueller-Hinton agar medium for bacteria and Sabouraud's agar medium for fungi. Briefly, 100 µl of the test bacteria/fungi were grown in 10 mL of fresh media until they reached a count of approximately10^8

cells/mL for bacteria or 10^5 cells/mL for fungi. All the newly synthesized compounds were weighed and dissolved in dimethyl sulfoxide to prepare extract stock solution. One hundred µL of each sample at 5 mg/mL was added to each well (10 mm diameter holes cut in the agar gel). The plates were incubated for 24-48 h at 37 °C (for bacteria and yeast) and for 48 h at 28 °C (for filamentous fungi). After incubation, the microorganism's growth was observed. The resulting inhibition zone diameters were measured in millimeters and used as criterion for the antimicrobial activity. The size of this clear zone is proportional to the inhibitory action of the compound under investigation. DMSO was used for dissolving the tested compounds thus used as solvent control and showed no inhibition zones, confirming that it has no influence on growth of the tested microorganisms. Positive controls were also performed using gentamycin as standard antibacterial drugs and ketoconazole as standard antifungal drug [38,39].

Figures of mean zone of inhibition of the newly synthesized pyrazolines

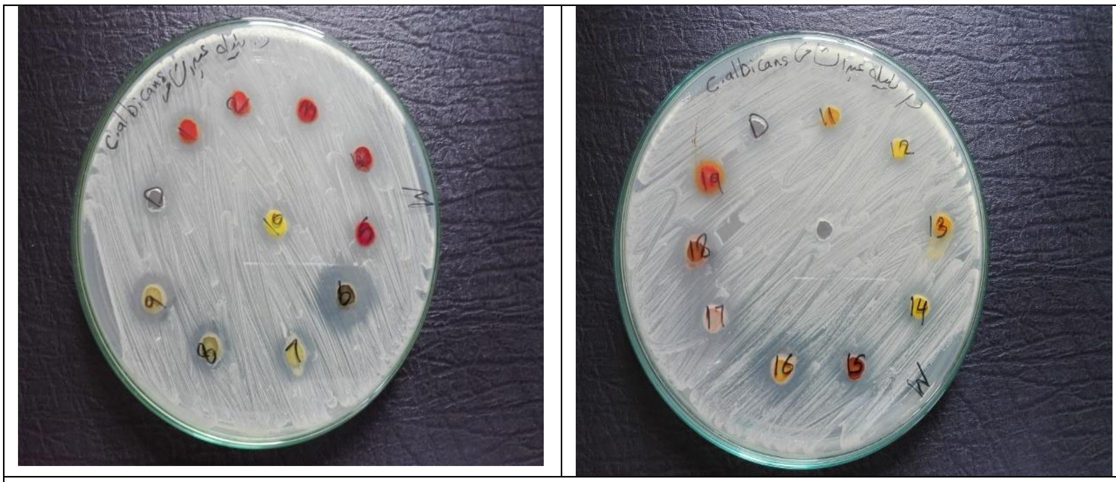

Fig. S1 Mean zone of inhibition of the newly synthesized pyrazolines tested against *Candida albicans* (RCMB 05036).

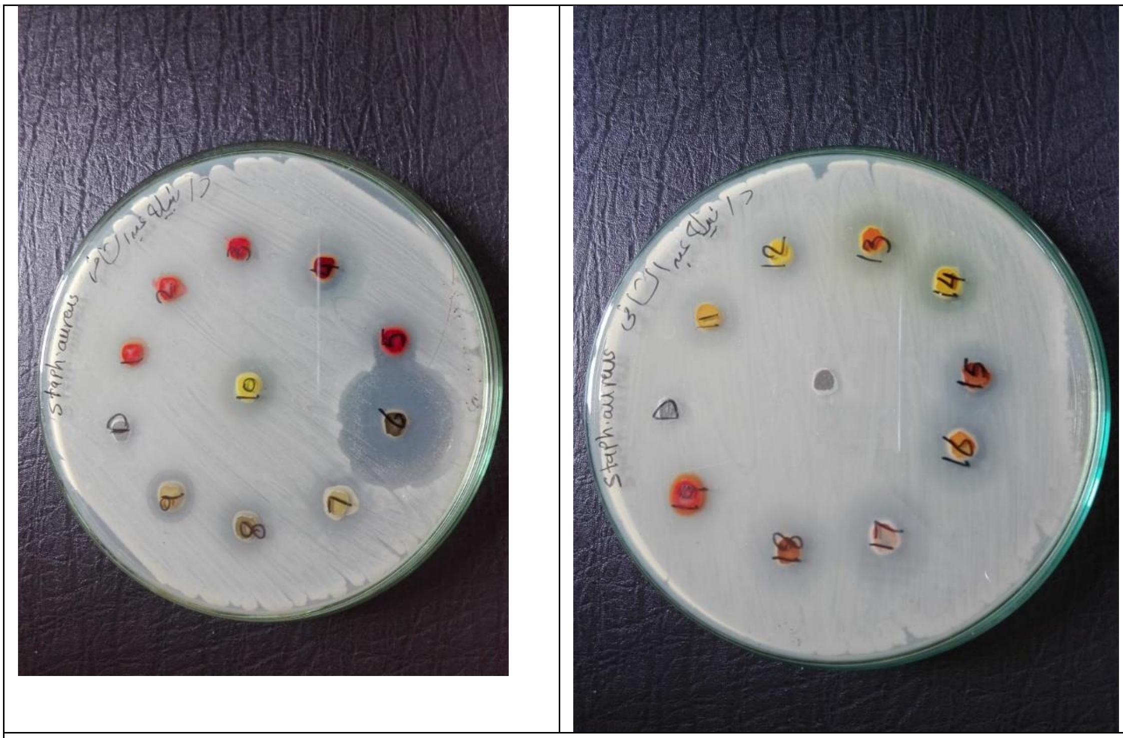

Fig S2. Mean zone of inhibition of the newly synthesized pyrazolines tested against *Staphylococcus aureus* (RCMB010010).

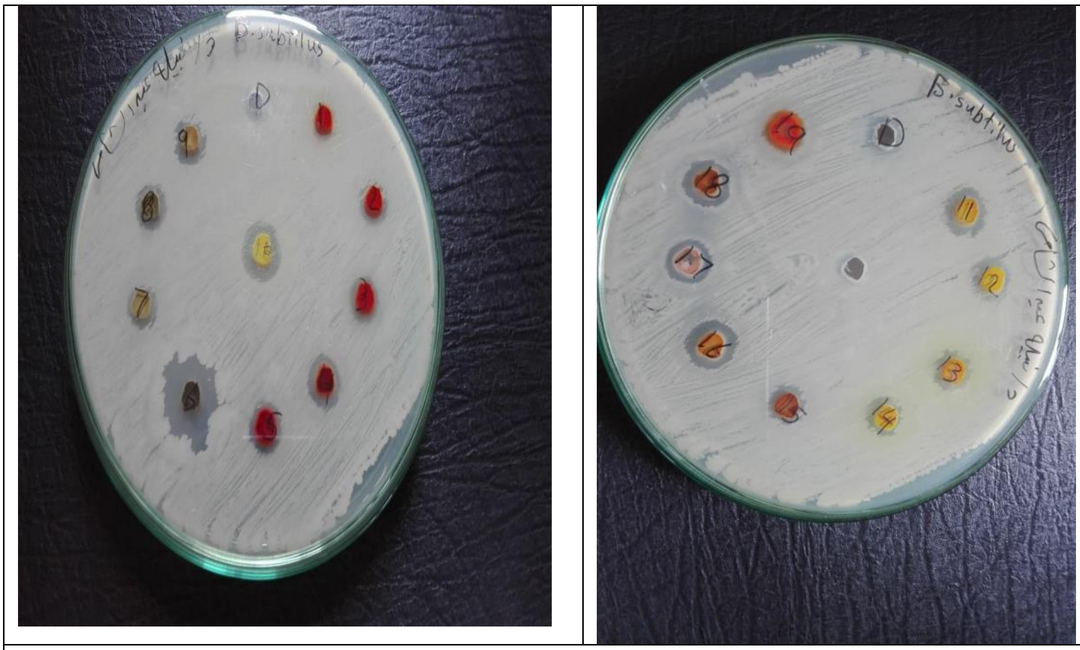

Fig S3. Mean zone of inhibition of the newly synthesized pyrazolines tested against *Bacillus subtilis* (RCMB 010067)

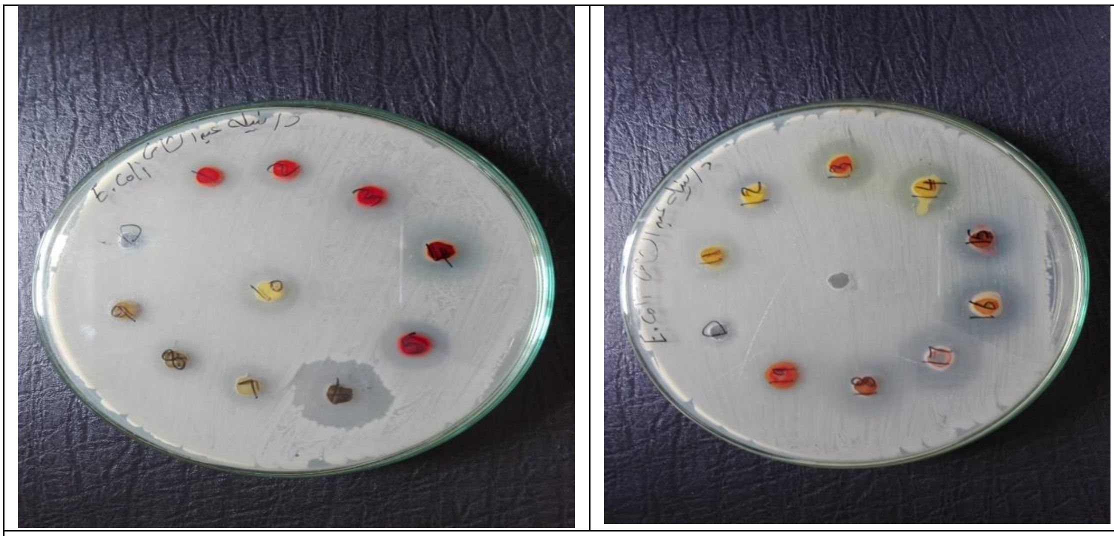

Fig S4. Mean zone of inhibition of the newly synthesized pyrazolines tested against *Escherichia coli* (RCMB 010052).

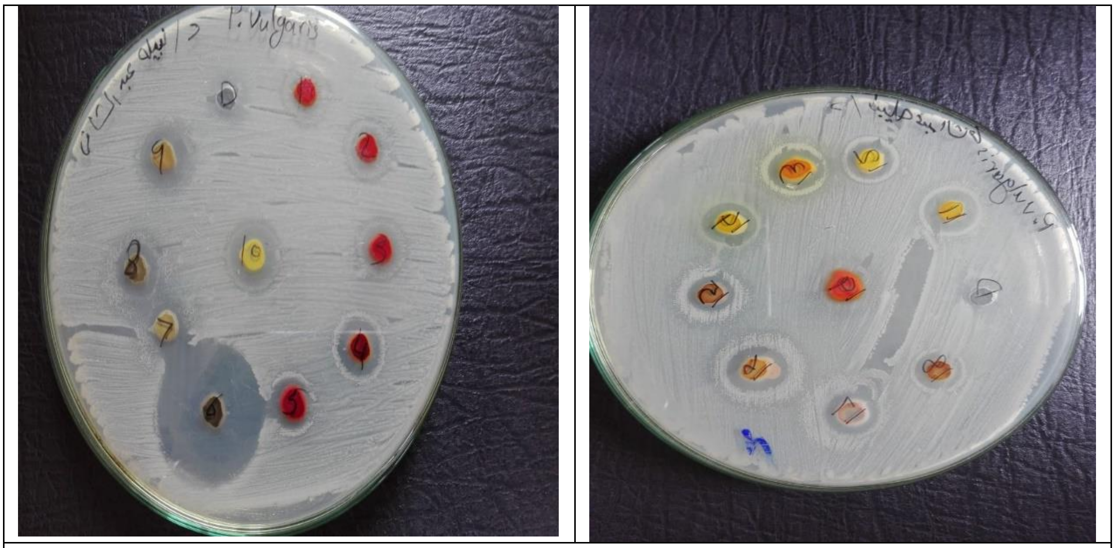

Fig S5. Mean zone of inhibition of the newly synthesized pyrazolines tested against *Proteus vulgaris* RCMB 004 (1) ATCC 13315

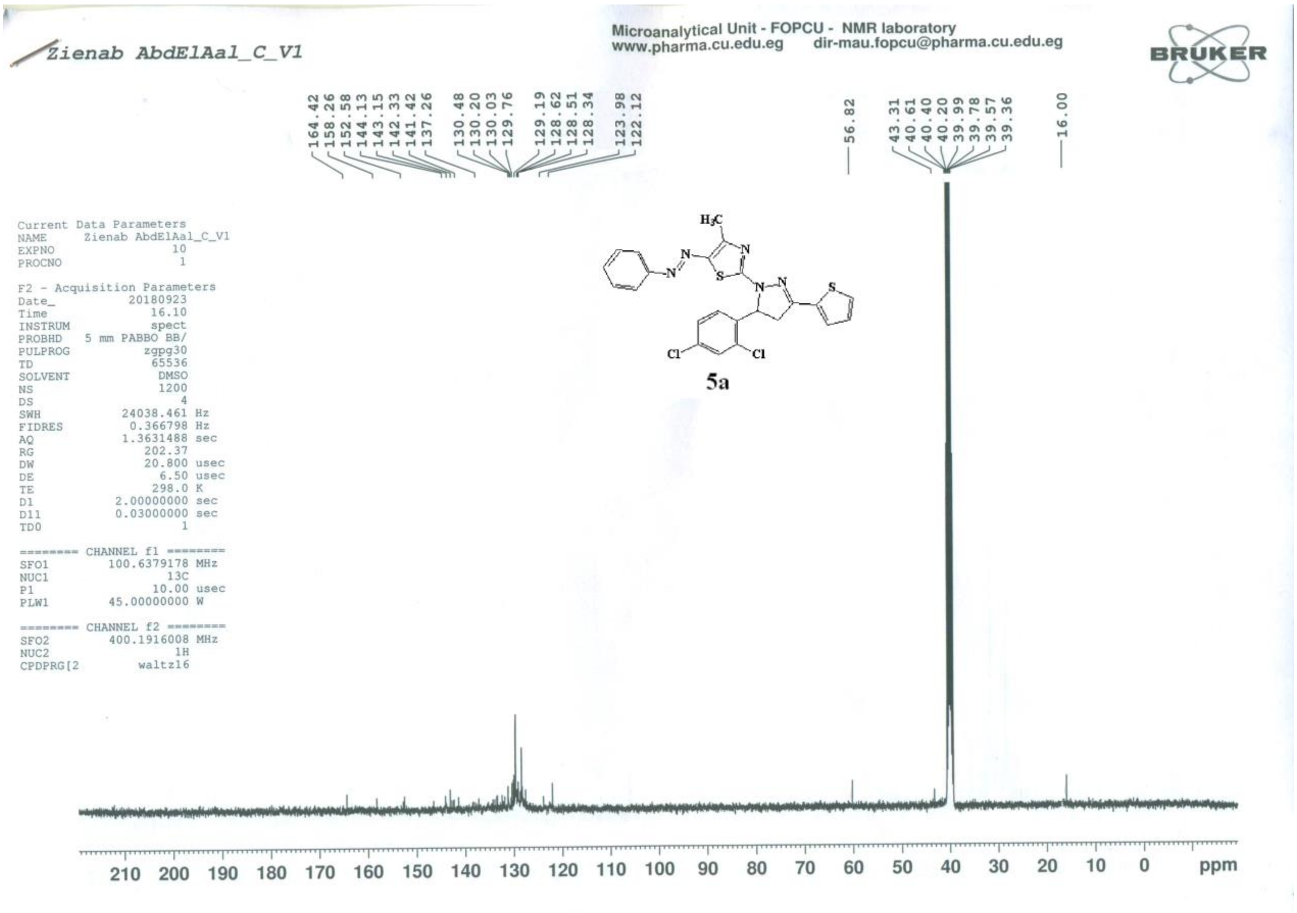

^{13}CNMR of compound **5a**

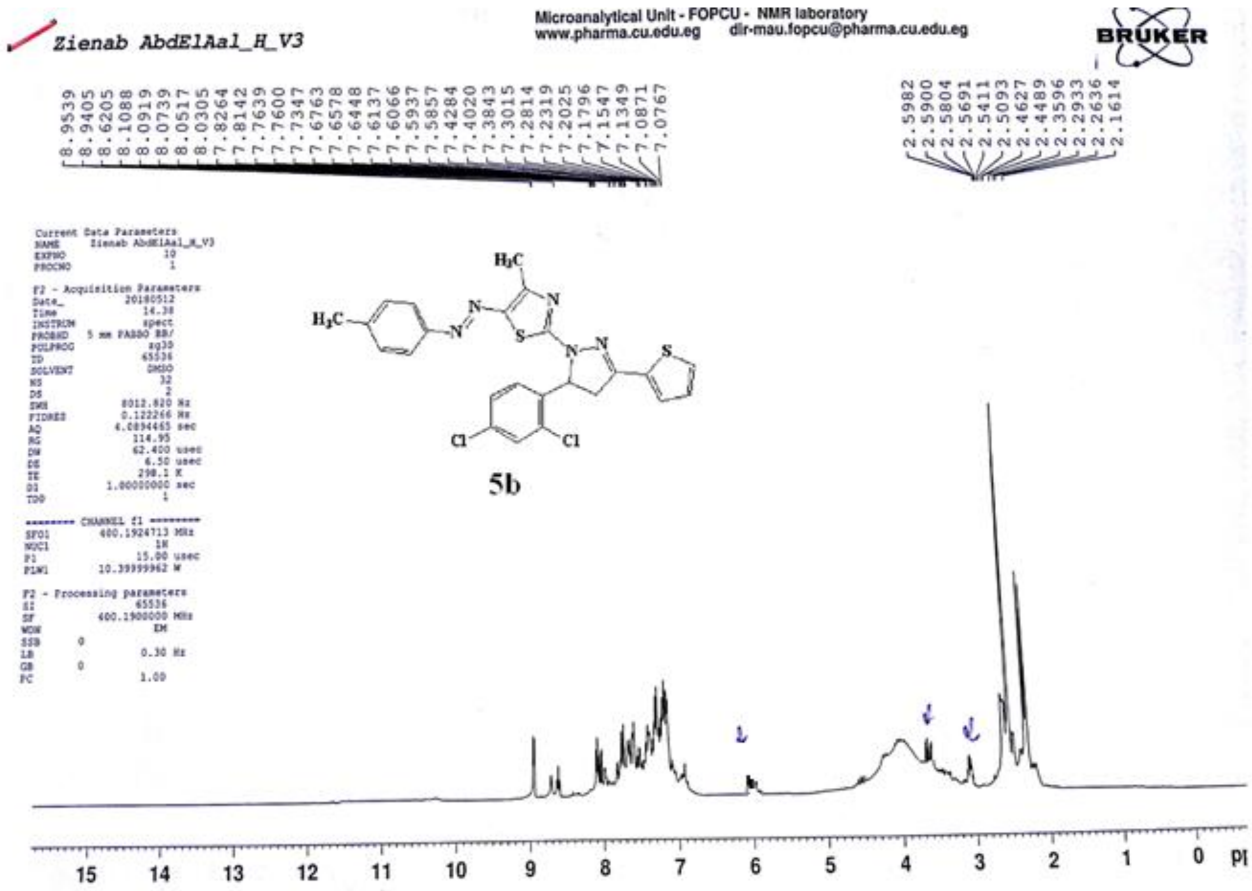

^{1}HNMR of compound **5b**

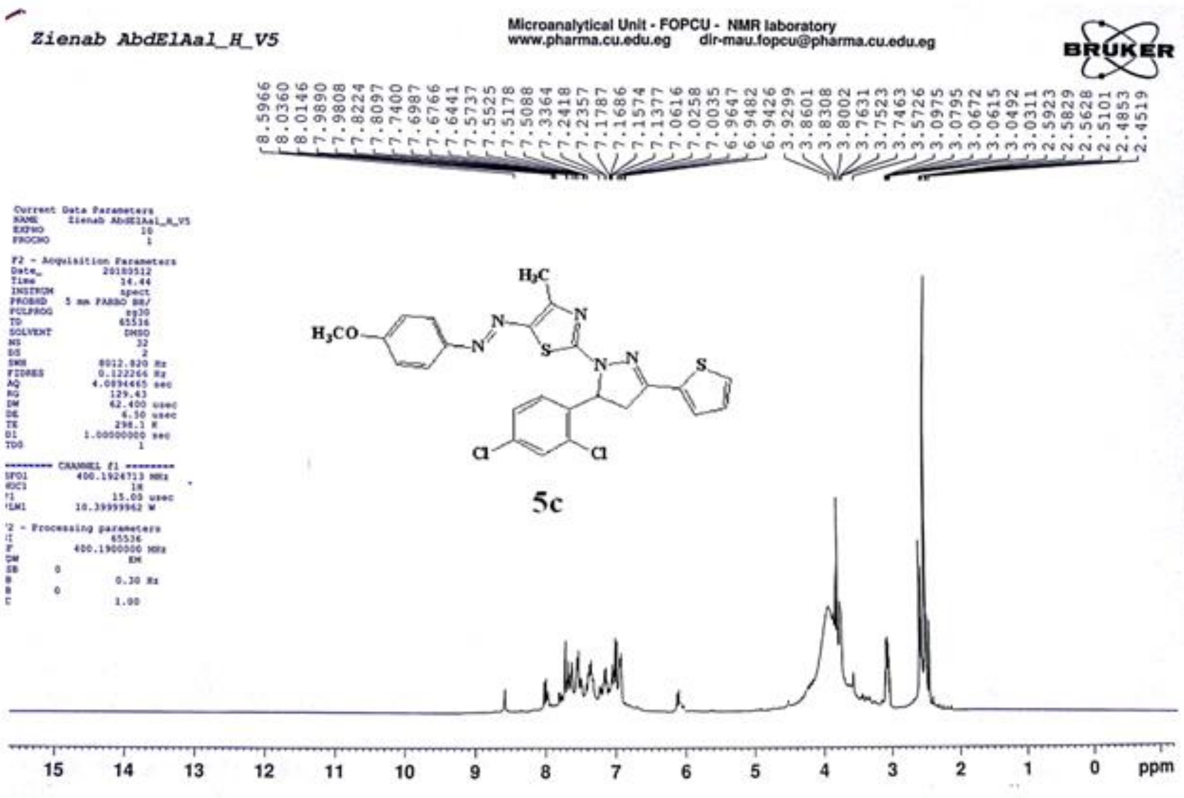

^{1}HNMR of compound **5c**

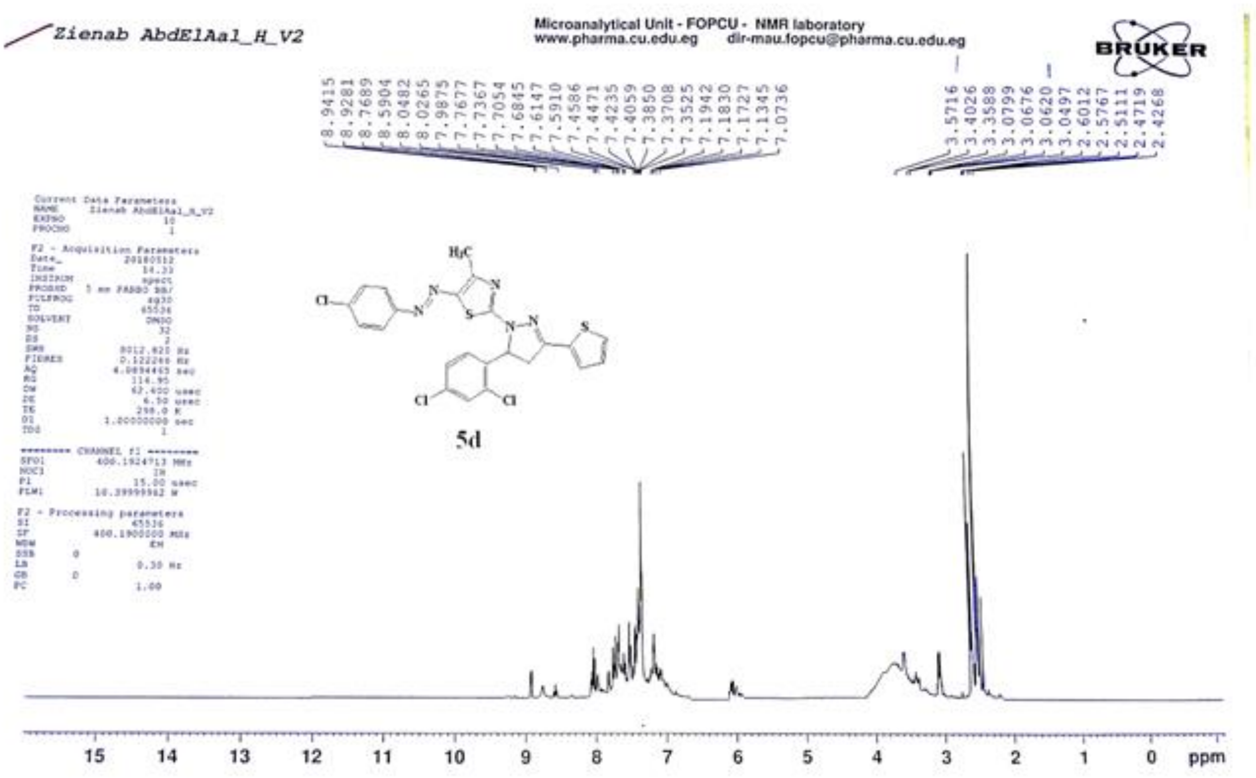

^{1}HNMR of compound **5d**

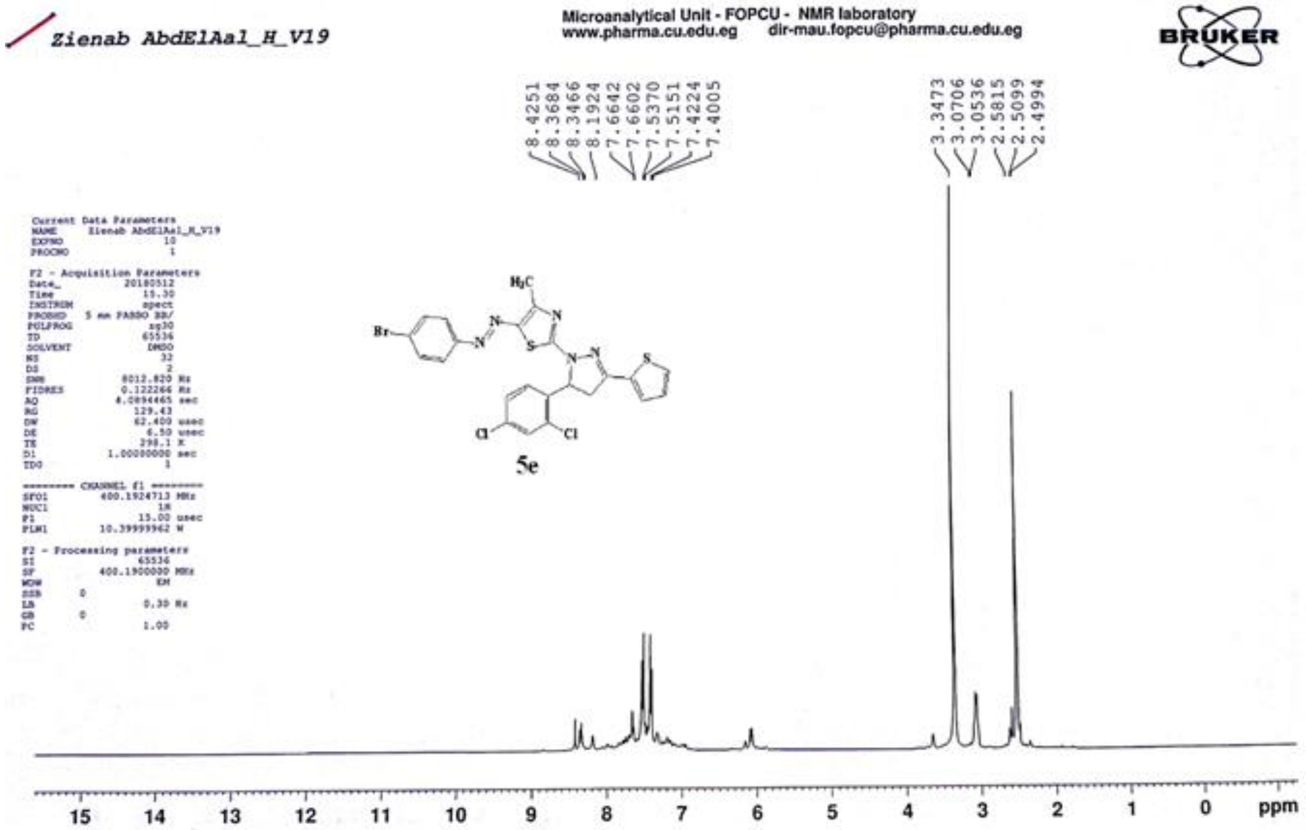

^{1}HNMR of compound **5e**

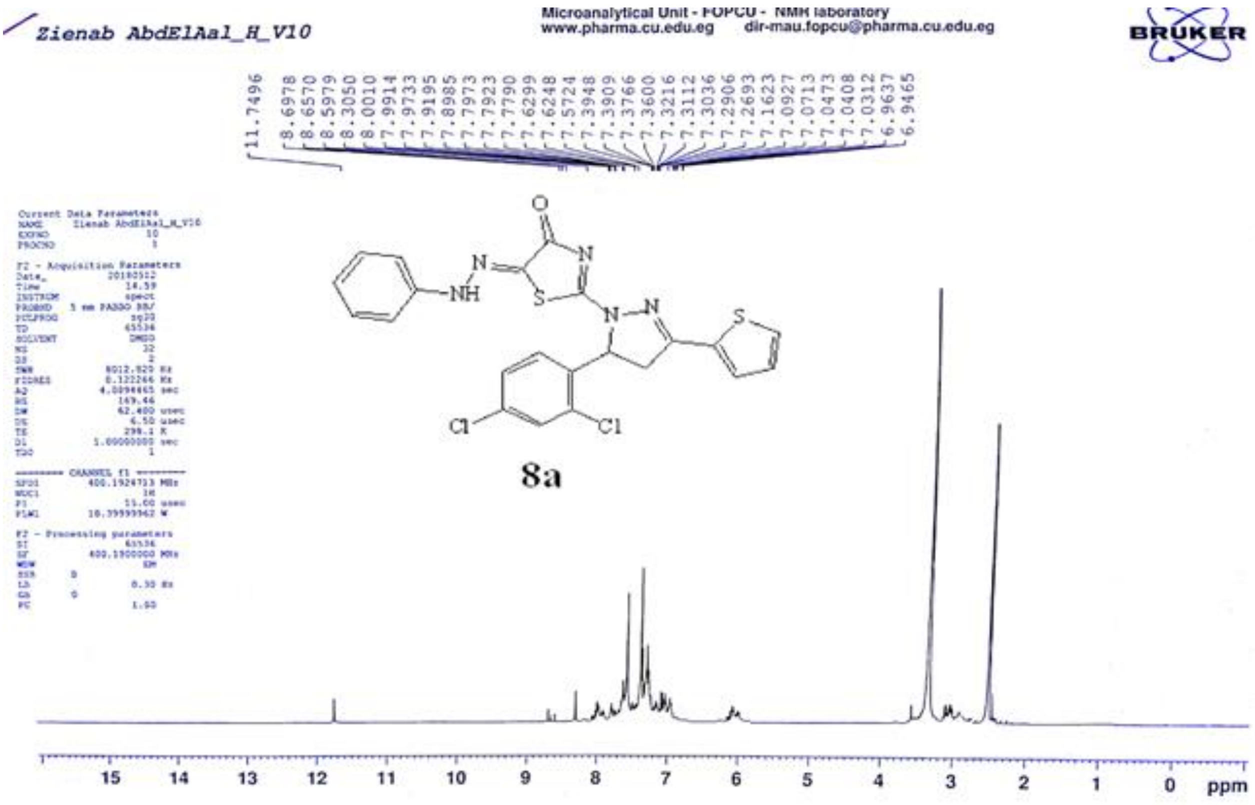

^{1}HNMR of compound **8a**

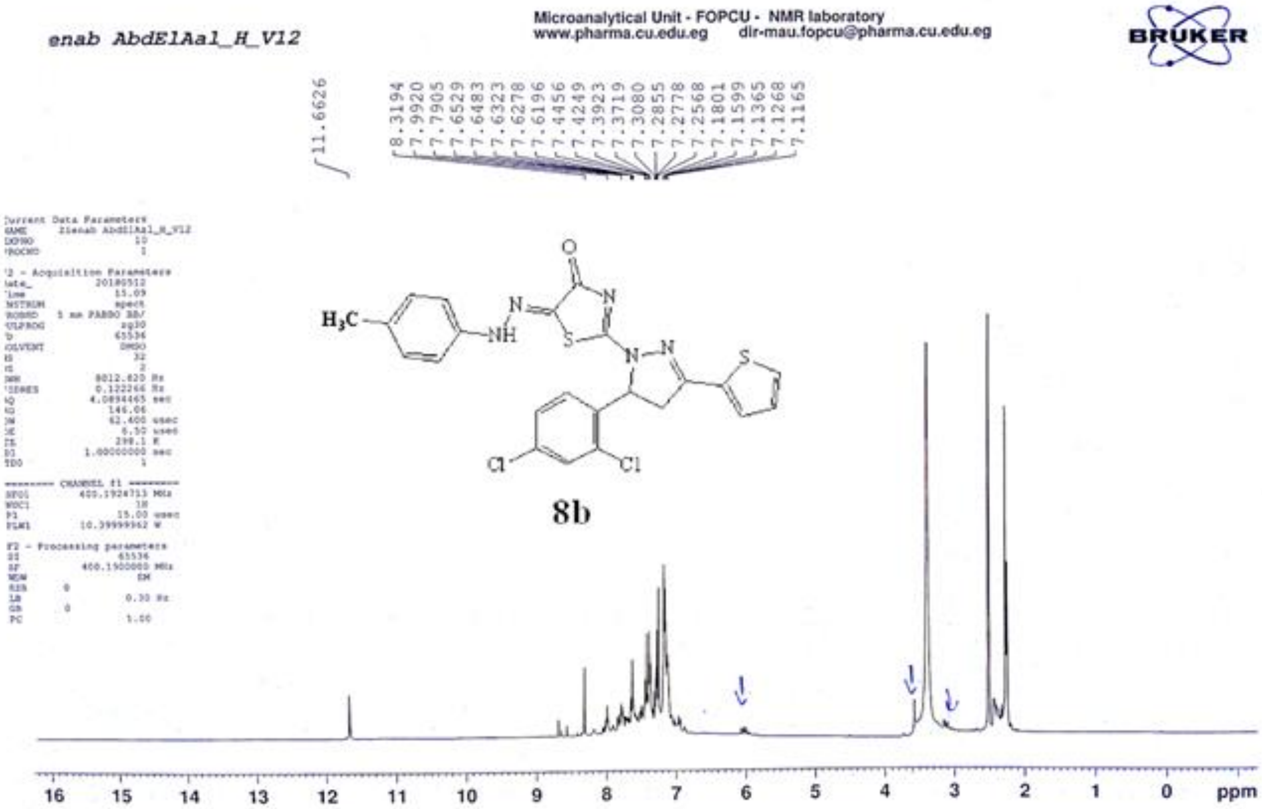

^{1}HNMR of compound **8b**

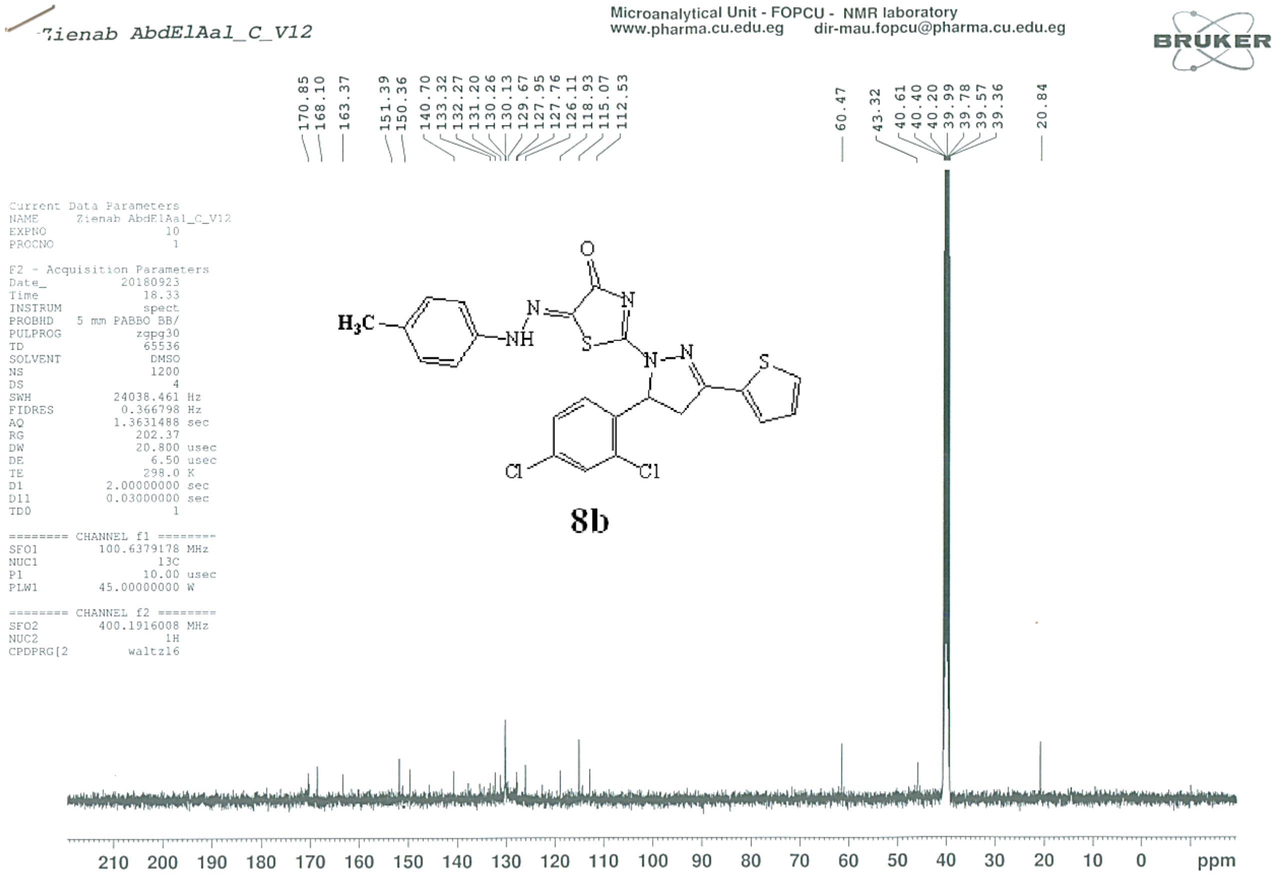

^{13}C-NMR of compound **8b**

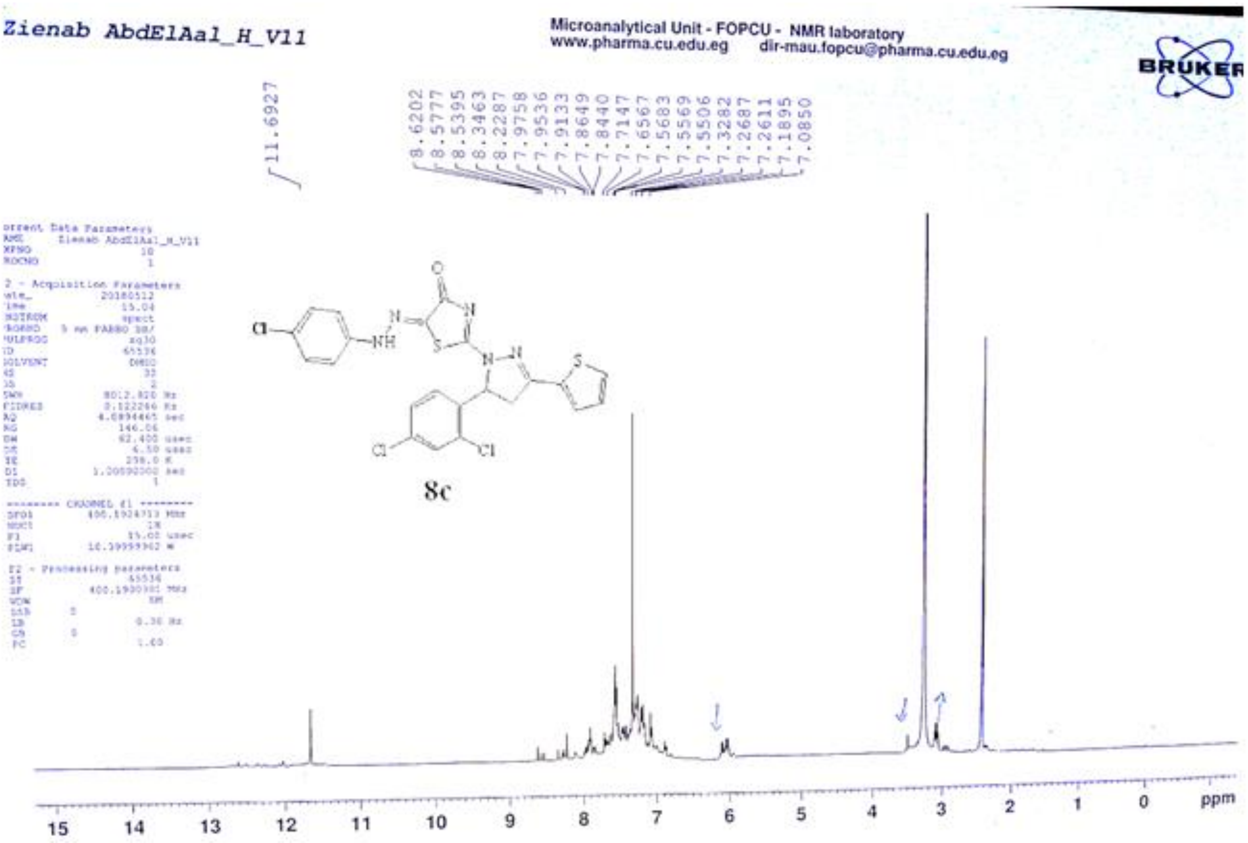

^{1}HNMR of compound **8c**

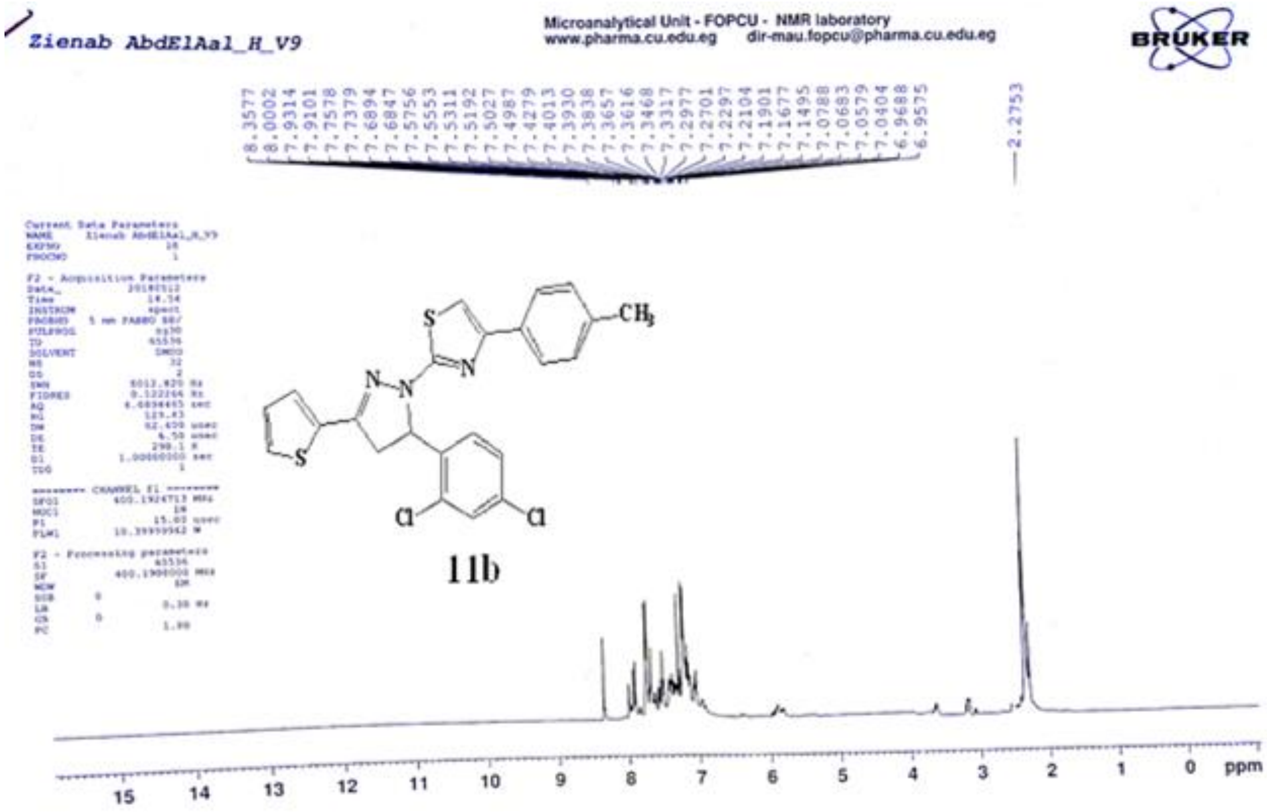

^{1}HNMR of compound **11b**

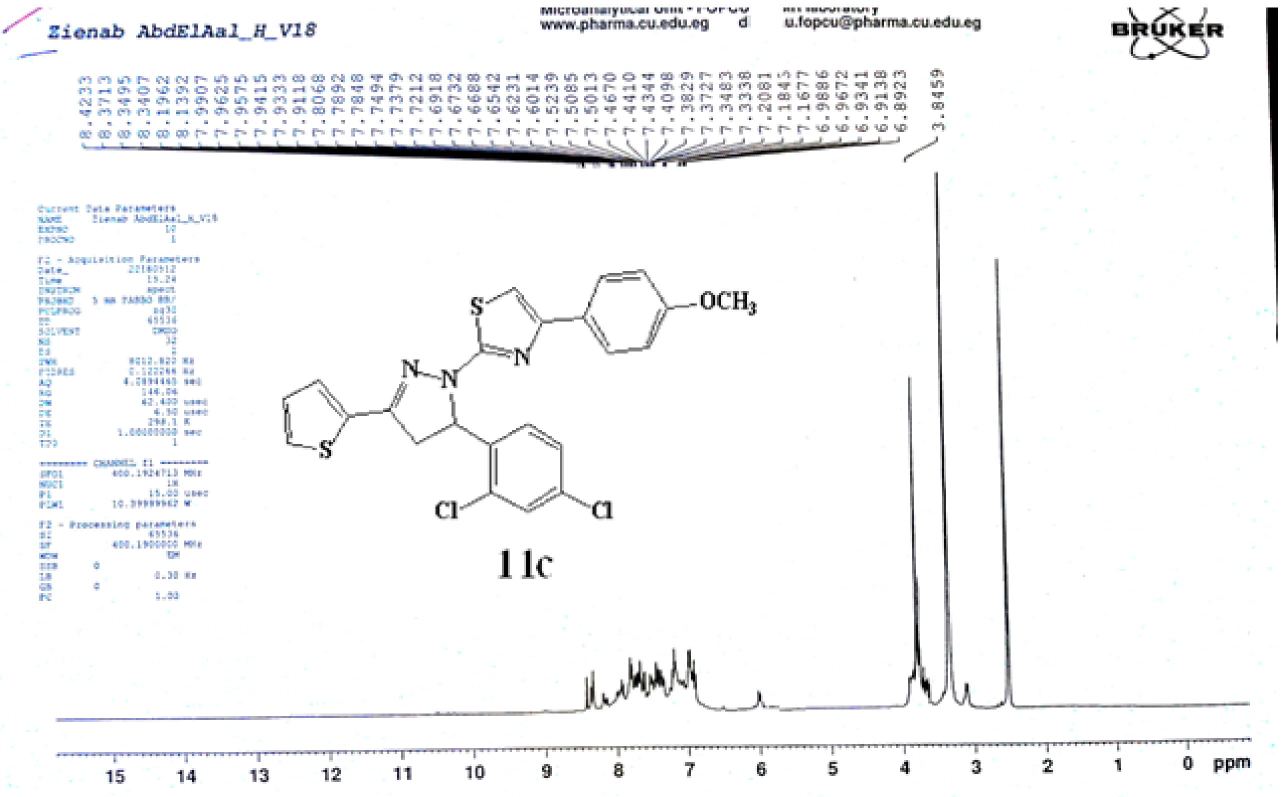

^{1}HNMR of compound **11c**

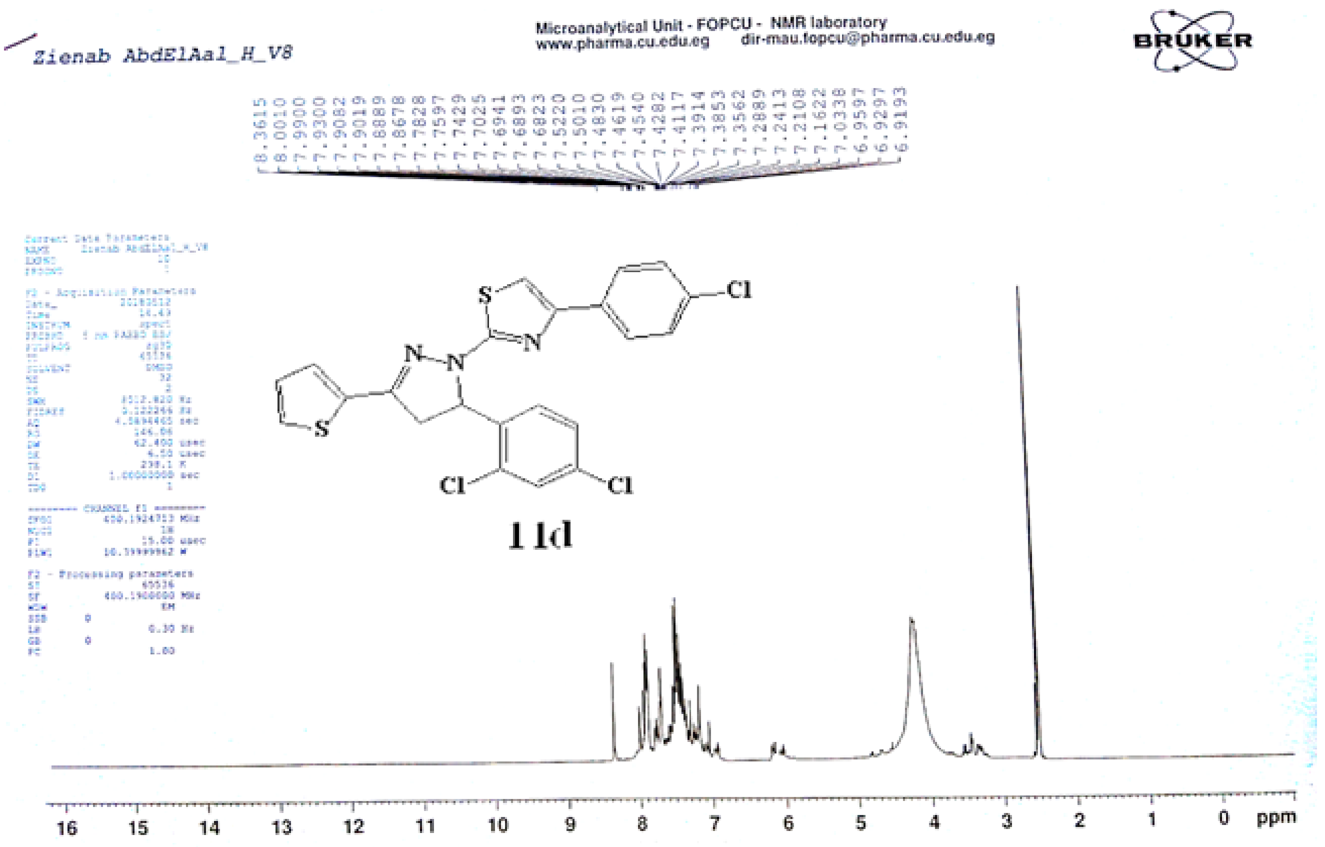

^{1}HNMR of compound **11d**

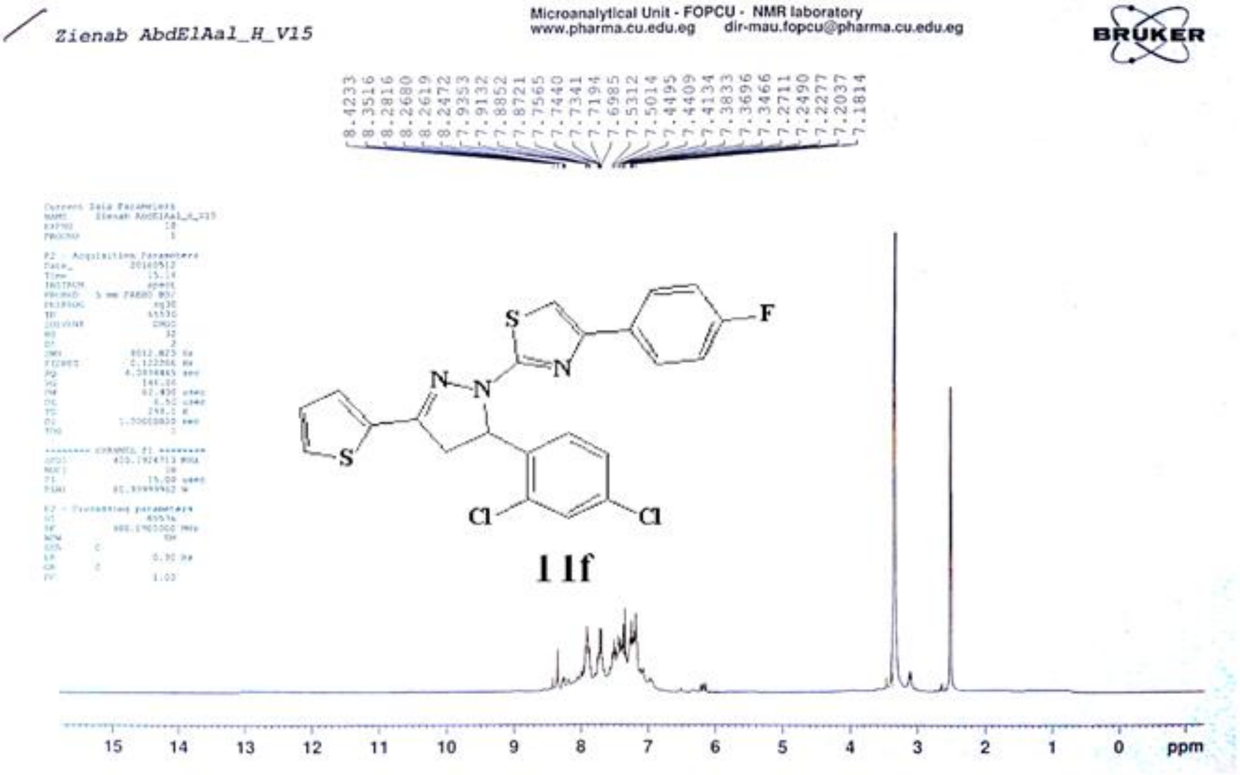

^{1}HNMR of compound **11f**
